# Inceptor binds to and directs insulin towards lysosomal degradation in β cells

**DOI:** 10.1038/s42255-024-01164-y

**Published:** 2024-11-25

**Authors:** Johanna Siehler, Sara Bilekova, Prisca Chapouton, Alessandro Dema, Pascal Albanese, Sem Tamara, Chirag Jain, Michael Sterr, Stephen J. Enos, Chunguang Chen, Chetna Malhotra, Adrian Villalba, Leopold Schomann, Sreya Bhattacharya, Jin Feng, Melis Akgün Canan, Federico Ribaudo, Ansarullah, Ingo Burtscher, Christin Ahlbrecht, Oliver Plettenburg, Thomas Kurth, Raphael Scharfmann, Stephan Speier, Richard A. Scheltema, Heiko Lickert

**Affiliations:** 1 Institute of Diabetes and Regeneration Research, Helmholtz Diabetes Center, Helmholtz Zentrum München, Neuherberg, Germany; 2 German Center for Diabetes Research (DZD), Neuherberg, Germany; 3 School of Medicine and Health, Technische Universität München, Munich, Germany; 4 Biomolecular Mass Spectrometry and Proteomics, Bijvoet Center for Biomolecular Research, Utrecht University, Utrecht, The Netherlands; 5 Utrecht Institute for Pharmaceutical Sciences, Utrecht University, Utrecht, Netherlands; 6 Proteomics Centre, Utrecht, The Netherlands; 7 Université Grenoble Alpes, IRIG, CEA, CNRS, INRAE, UMR 5168, 38000, Grenoble, France; 8 Université Grenoble Alpes, INSERM, IRIG, CEA, UA13 BGE, CNRS, CEA, FR2048 ProFI, 38000, Grenoble, France; 9 Institute of Physiology, Faculty of Medicine Carl Gustav Carus, Dresden University of Technology, Dresden, Germany; 10 Paul Langerhans Institute Dresden (PLID) of the Helmholtz Zentrum München at the University Clinic Carl Gustav Carus of Dresden University of Technology, Dresden, Germany; 11 TUM School of Life Sciences, Technische Universität München, Freising, Germany; 12 Université Paris Cité, Institut Cochin, CNRS, INSERM, Paris, France; 13 Institute for Medicinal Chemistry, Molecular Targets and Therapeutics Center, Helmholtz Zentrum München, Neuherberg, Germany; 14 Institute of Organic Chemistry (BMWZ) and Laboratory for Nano- and Quantum Engineering (LNQE), Center of Biomolecular Research, Leibniz, University Hannover, Hannover, Germany; 15 Institute of Lung Health (ILH), Giessen, Germany; 16 Center for Molecular and Cellular Bioengineering (CMCB), Technology Platform, Core Facility Electron Microscopy and Histology, Dresden University of Technology, Dresden, Germany; 17 Center for Proteome, Research and Department of Biochemistry, University of Liverpool, Liverpool, UK

**Keywords:** Insulin signalling, Hormones, Metabolism, Stem cells

## Abstract

Blunted first-phase insulin secretion and insulin deficiency are indicators of β cell dysfunction and diabetes manifestation. Therefore, insights into molecular mechanisms that regulate insulin homeostasis might provide entry sites to replenish insulin content and restore β cell function. Here, we identify the insulin inhibitory receptor (inceptor; encoded by the gene *IIR/ELAPOR1*) as an insulin-binding receptor that regulates insulin stores by lysosomal degradation. Using human induced pluripotent stem cell (SC)-derived islets, we show that *IIR* knockout (KO) results in enhanced SC β cell differentiation and survival. Strikingly, extended in vitro culture of *IIR* KO SC β cells leads to greatly increased insulin content and glucose-stimulated insulin secretion (GSIS). We find that inceptor localizes to clathrin-coated vesicles close to the plasma membrane and in the *trans*-Golgi network as well as in secretory granules, where it acts as a sorting receptor to direct proinsulin and insulin towards lysosomal degradation. Targeting inceptor using a monoclonal antibody increases proinsulin and insulin content and improves SC β cell GSIS. Altogether, our findings reveal the basic mechanisms of β cell insulin turnover and identify inceptor as an insulin degradation receptor.

## Abstract (web summary)

The insulin inhibitory receptor (inceptor) is found to bind to insulin and to regulate insulin stores by directing proinsulin and insulin towards lysosomal degradation.

## Main

Pancreatic β cells are highly specialized secretory cells that produce, process, store and secrete the glucoregulatory hormone insulin. Sorting proinsulin from the *trans*-Golgi network (TGN) to secretory granules (SGs) is critical for its conversion into insulin and the formation of insulin SGs, which are essential for the tight regulation of glucose homeostasis in mammals. As early as the 1980s, it was suspected that specialized clathrin-coated membranes associated with the Golgi apparatus ‘ship’ granule proteins and proinsulin by membrane-bound receptors into SGs—analogous to ligands internalized by clathrin-mediated endocytosis of receptors at the plasma membrane^1^. Despite initial excitement that carboxypeptidase E could be a prohormone and proinsulin sorting receptor^2^, this idea could not be confirmed^3^, and proinsulin sorting receptor(s) have not been identified until recently. Currently, there are models for selective and non-selective entry mechanisms of proinsulin into SGs by ‘sorting by entry’^4–7^. Interestingly, proteolytic enzymes and other proteins are retrieved from immature SGs (iSGs) by mannose-6-phosphate receptors (M6PRs) in clathrin-coated vesicles (CCVs) by a ‘sorting by exit’ mechanism^8^. At the same time, proinsulin and C-peptide can exit the SGs in this process passively in the fluid phase and be further directed to endosomal sorting and/or constitutive-like secretion^9,10^. Around 99% of insulin is secreted through the regulated secretory pathway rather than constitutively^11^; however, whether control mechanisms to prevent proinsulin secretion exist is unknown.

SG degradation has been observed in β cells under growth conditions^12–14^, starvation^15^ or stress^16^, allowing clearance of older SGs and preventing uncontrolled insulin secretion, endoplasmic reticulum stress or lack of nutrients. Specifically, SGs are delivered to lysosomes by two distinct mechanisms: firstly, through macroautophagy, in which granules encapsulated in double-membraned autophagosomes fuse with lysosomes^14,17^; and secondly, through crinophagy, in which SGs directly fuse with lysosomes^13,18^. Importantly, SG homeostasis imbalance resulting from the failure of the degradation processes has been reported in diabetic and degranulated β cells^16,19–23^. For example, β cell failure is accompanied by stress-induced nascent granule degradation^16^. These processes, however, describe the degradation of intact iSGs and mature SGs (mSGs). Alternatively, proinsulin could be degraded by a ‘sorting by exit’ mechanism, in which retrieval and transport of proinsulin from iSGs to the lysosome occurs while the SG stays intact and further matures. Despite considerable interest and the description of RILP-dependent and VAMP4-dependent lysosomal targeting of (pro)insulin^24,25^, it is unknown whether a targeted ‘sorting by exit’ mechanism exists at the site of nascent SG formation.

Inceptor (also known as ELAPOR1/KIAA1324/EIG121) is a transmembrane protein that functions in lysosomal degradation, autophagy, zymogenic granule maturation and acrosome formation^26–28^. We recently described inceptor as a negative regulator of insulin receptor (INSR) and insulin-like growth factor 1 receptor (IGF1R) signalling^29^. It facilitates clathrin-mediated endocytosis of the INSR and IGF1R, desensitizing β cells to autocrine and paracrine insulin signalling^29^. Furthermore, we showed that the pancreas of *Iir*^–/–^ mice is characterized by elevated insulin levels^29^, the mechanism of which has not yet been described. Inceptor shares structural similarities and predominant subcellular localization with the cation-independent and cation-dependent M6PRs (CI-M6PR and CD-M6PR, respectively), which shuttle cargo from the TGN through sorting endosomes to lysosomes^30^. Inceptor also contains the lysosomal targeting signal YXXΦ, in which Φ indicates a bulky hydrophobic amino acid and X indicates any amino acid^29^. Here, we show that like the CD-M6PR and CI-M6PR, inceptor has an additional function as an intracellular lysosomal sorting receptor. We provide evidence that inceptor is a receptor for proinsulin and insulin and it routes them towards lysosomes for degradation. Accordingly, β cells derived from human *Iir*^–/–^ stem cells (SC β cells) and SC β cells treated with monoclonal antibodies (mABs) targeting inceptor were characterized by elevated proinsulin and insulin levels, mimicking the phenotype of the β cells from *Iir*^–/–^ mice^29^. In summary, our study suggests a previously unknown function of inceptor in insulin homeostasis.

## Results

### Inceptor regulates SC β cell differentiation and survival

**Fig. 1:**
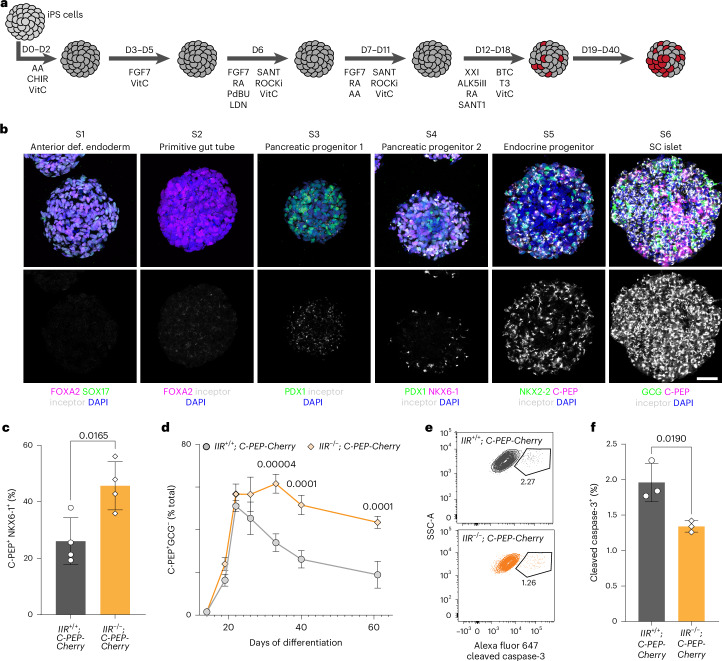
*IIR* KO improves SC β cell differentiation and survival. **a**, Overview of the applied six-stage (S1–S6) differentiation protocol. D, day. **b**, Expression of stage-specific markers (top panels) and inceptor expression (bottom panels) from S1 to S6. Maximum intensity projections are shown. Scale bar, 50 µm (*n* = 3). **c**, Flow cytometry quantification of C-PEP^+^NKX6-1^+^ SC β cells at the end of S6 at differentiation D40 (*n* = 4, mean ± s.d., unpaired two-tailed *t*-test). **d**, Flow cytometry quantification of C-PEP^+^ and glucagon (GCG)^−^ SC β cells during S5 and extended S6 culture until D61 (*n* = 3, mean ± s.d., two-way ANOVA followed by Šidák’s multiple comparisons test). **e**,**f**, Representative flow cytometry analysis (**e**) and quantification (**f**) of cleaved caspase-3^+^ cells at D40 in the C-PEP^+^ SC β cell subpopulation (in **f**, *n* = 3, mean ± s.d., unpaired two-tailed *t*-test). Source data

**Extended Data Fig. 1:**
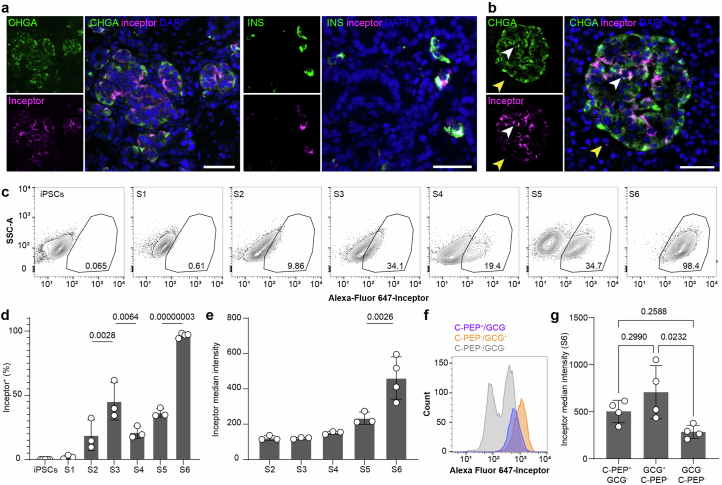
Inceptor is expressed throughout human β cell development *in vivo* and SC β cell differentiation *in vitro.* **a, b**, Immunostaining of human foetal pancreas at 11.4 weeks of development (**a**) and human adult pancreas (**b**) for inceptor, Chromogranin A (CHGA) and insulin; the white arrow indicates a CHGA/inceptor double-positive cell, the yellow arrow indicates inceptor expression in surrounding exocrine tissue. Scale bar, 50 µm (*n* = 1). **c**, Representative plots of the flow cytometry analysis of inceptor expression during the differentiation of iPS cells towards SC-islets. **d, e**, Flow cytometry-based quantification of the fraction of inceptor-positive cells (**d**) and the fluorescent intensity of inceptor considering only inceptor-positive cells (**e**) (iPS cells, *n* = 5; S1 – S5, *n* = 3; S6, *n* = 4; mean ± s.d.; one-way ANOVA followed by Tukey’s multiple comparisons test). **f, g**, Representative histogram (**f**) and quantification (**g**) of the flow cytometry analysis of hormone expression and inceptor fluorescent intensity at S6. Only inceptor-positive cells within the SC β cell (C-PEP^+^GCG^−^), SC α cell (C-PEP^−^GCG^+^) or hormone-negative cell (C-PEP^−^GCG^−^) population were considered (**g**, *n* = 4, mean ± s.d., one-way ANOVA followed by Tukey’s multiple comparisons test). Source data

To recapitulate and translate our previous in vivo findings from mouse to human, we studied the function of inceptor during the stage-wise differentiation of human induced pluripotent stem cells (iPS cells) towards SC islets, which mimics key processes of in vivo pancreas and islet development (Fig. 1a)^31,32^. During human foetal pancreas development, a subpopulation of chromogranin A (CHGA)-positive endocrine progenitors and insulin-positive β cells at 11.4 weeks express inceptor (Extended Data Fig. 1a). In the adult human pancreas, inceptor is present in the endocrine cells of the islet and, to a lower extent, in the surrounding exocrine tissue (Extended Data Fig. 1b). In vitro, inceptor expression faithfully recapitulates the in vivo expression pattern, with onset of expression in pancreatic progenitors followed by strong upregulation in endocrine progenitors and hormone-positive SC α cells and SC β cells formed during the final iPS cell differentiation stage (Fig. 1b and Extended Data Fig. 1c–g).

**Extended Data Fig. 2:**
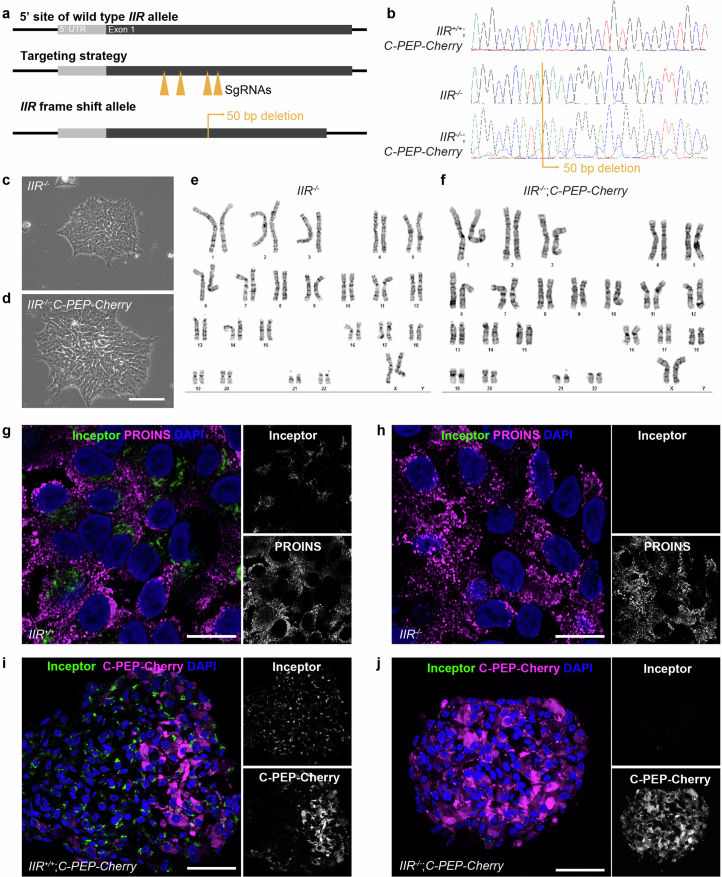
Generation of *IIR* KO iPS cell lines. **a**, Schematic representation of the CRISPR/Cas9 targeting strategy to introduce an *IIR* deletion by frame shift mutation in exon 1. **b**, Sanger sequencing of exon 1 of *IIR,* demonstrating the deletion of 50 bp in the lines *IIR*^*−/−*^ and *IIR*^*−/−*^*;C-PEP*^*−*^*Cherry*. **c, d**, *IIR*^*−/−*^ (**c**) and *IIR*^*−/−*^*; C-PEP-Cherry* (**d**) iPS cell clones, showing pluripotent morphology. Scale bar, 100 µm (*n* = 3). **e, f**, Unaltered, female karyotypes of the *IIR*^*−/−*^ (**e**) and *IIR*^*−/−*^*;C-PEP-Cherry* (**f**) iPS cell lines. **g-j**, Immunostainings of S6 SC-islets showing expression of inceptor in *IIR*^*+/+*^ (**g**) and *IIR*^*+/+*^*;C-PEP-Cherry* (**i**) cells and greatly reduced inceptor expression in *IIR*^*−/−*^ (**h**) and *IIR*^*−/−*^*;C-PEP-Cherry* (**j**) cells. Scale bars, 10 µm (**g, h**), 50 μm (**i, j**), (*n* = 3).

**Extended Data Fig. 3:**
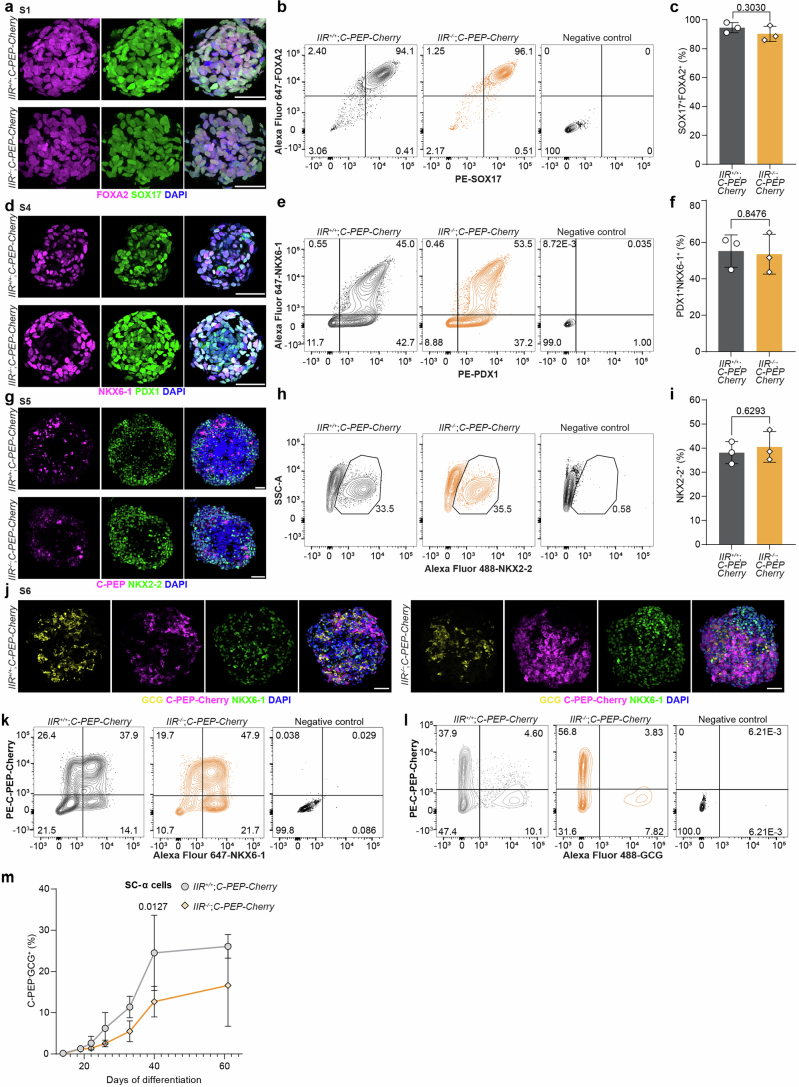
*IIR* KO iPS cells show unaltered pancreatic progenitor differentiation. **a-c**, Representative confocal images (**a**), flow cytometry plots (**b**) and quantification (**c**) of the analysis of FOXA2^+^SOX17^+^ cells at the anterior definitive endoderm stage (S1) (*n* = 3, mean ± s.d., unpaired two-tailed *t*-test). **d-f**, Representative confocal images (**d**), flow cytometry plots (**e**) and quantification (**f**) of the analysis of PDX1^+^NKX6-1^+^ cells at the pancreatic progenitor stage 2 (S4) (*n* = 3, mean ± s.d., unpaired two-tailed *t*-test). **g-i**, Representative confocal images (**g**), flow cytometry plots (**h**) and quantification (**i**) of the analysis of NKX2-2^+^ cells at the endocrine progenitor stage (S5) (*n* = 3, mean ± s.d., unpaired two-tailed *t*-test). **j-l**, Representative confocal images (**j**) and flow cytometry plots (**k, l**) of the analysis of GCG^+^ SC α cells and C-PEP^+^NKX6-1^+^ SC β cells at the SC-islet stage (S6, D30) (*n* = 3). **a, d, g, j**, Maximum intensity projections. Scale bars, 50 μm. **m**, Flow cytometry quantification of C-PEP^-^GCG^+^ SC α cells during S5 and extended S6 culture until D61 (*n* = 3, mean ± s.d., two-way ANOVA followed by Sidak’s multiple comparisons test). Source data

**Extended Data Fig. 4:**
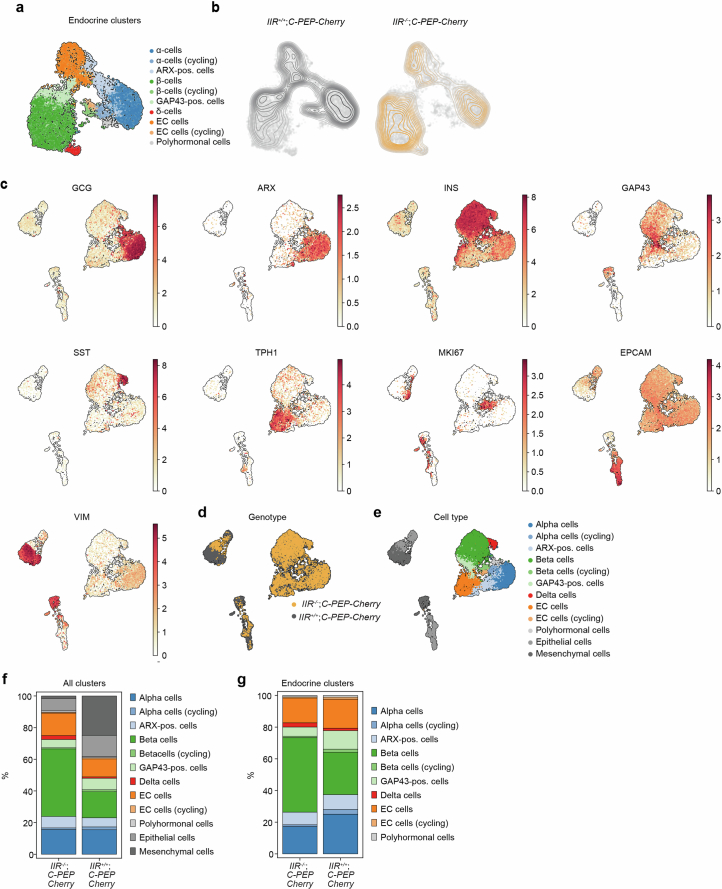
Profiling *IIR* KO SC islets by scRNA sequencing. **a-g**, scRNA sequencing analysis of *IIR*^+/+^;C-PEP-Cherry and *IIR*^-/-^;C-PEP-Cherry SC-islets on D40. UMAP projections show identified endocrine clusters (**a**), cell distribution according to the genotype of the sample (**b**), clusters with corresponding marker gene expression (**c**), integrated cell distribution according to genotype (**d**) and assigned clusters (**e**); **f, g**, fraction of cells assigned to the respective endocrine clusters, considering all cells (**f**) or endocrine cells only (**g**). Source data

We have previously shown that whole-body *Iir*^−/−^ mice had an increased β cell mass at birth^29^. To determine the relevance of inceptor in human β cells, we analysed SC islets derived from a control iPS cell line (*IIR*^+/+^)^33^, an inceptor KO line (*IIR*^−/−^), a control C-peptide (C-PEP)–Cherry reporter iPS cell line (*IIR*^+/+^; *C-PEP-Cherry*)^34^ and an inceptor KO C-PEP–Cherry iPS cell line (*IIR*^−/−^; *C-PEP-Cherry*). The KO lines generated by CRISPR–Cas9 gene targeting (see Methods and Extended Data Fig. 2a,b) displayed a pluripotent morphology (Extended Data Fig. 2c,d) and an unaltered female karyotype (Extended Data Fig. 2e,f). Importantly, inceptor protein expression as determined by immunostaining was greatly reduced in *IIR*^−/−^ and *IIR*^−/−^; *C-PEP-Cherry* SC islets (Extended Data Fig. 2g–j). First, we compared the differentiation efficiencies of both C-PEP–Cherry iPS cell lines at key stages of in vitro differentiation towards SC islets. Both lines generated similar percentages of anterior definitive endoderm (S1; Extended Data Fig. 3a–c), pancreatic progenitors (S4; Extended Data Fig. 3d–f) and endocrine progenitors (S5; Extended Data Fig. 3g–i). As expected from the strong expression of inceptor in later stages of endocrine differentiation, inceptor is dispensable for pancreatic progenitor cell formation. Intriguingly, we found an increased percentage of SC β cells in *IIR*^−/^^−^; *C-PEP-Cherry* SC islets at S6 (Fig. 1c and Extended Data Fig. 3j,k). Notably, *IIR*^−/−^; *C-PEP-Cherry* SC islets maintained SC β cell numbers in extended S6 cultures, hinting towards improved SC β cell survival (Fig. 1d and Extended Data Fig. 3l). This is supported by the reduction of the percentage of apoptotic, cleaved caspase-3-positive SC β cells within *IIR*^−/−^; *C-PEP-Cherry* SC islets (Fig. 1e,f). In contrast to SC β cells, SC α cell percentages were reduced in the *IIR*^−/−^; *C-PEP-Cherry* line (Extended Data Fig. 3l,m). To further characterize the *IIR*^+/+^; *C-PEP-Cherry* and *IIR*^−/^^−^; *C-PEP-Cherry* SC islets, we profiled them by single-cell RNA sequencing (scRNA-seq). Cluster annotation and analysis revealed a comparable SC islet cell type composition and confirmed the increase of SC β cell and decrease of SC α cell percentages in *IIR*^−/−^; *C-PEP-Cherry* SC islets (Extended Data Fig. 4). Thus, the lack of inceptor improves SC β cell differentiation and survival during endocrine cell formation and extended culture in vitro.

### Inceptor regulates insulin turnover

**Fig. 2:**
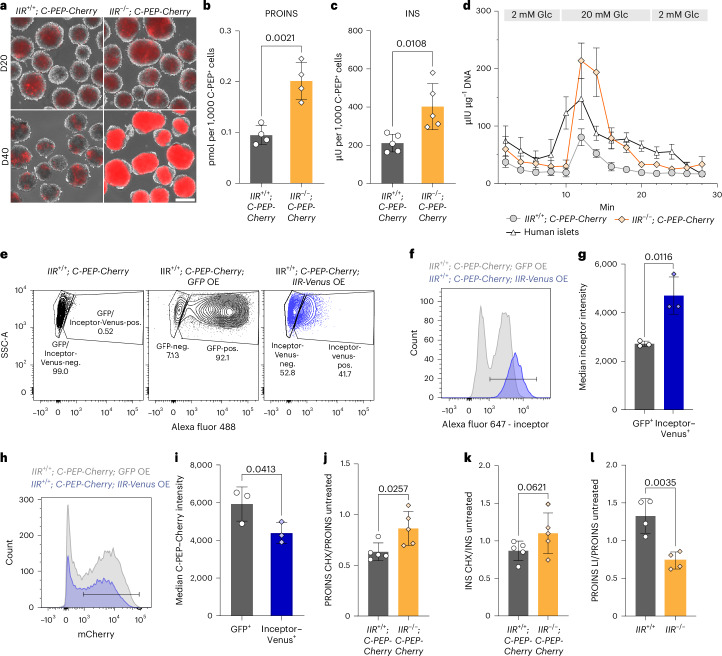
Inceptor is a negative regulator of insulin homeostasis, mediating proinsulin degradation. **a**, C-PEP–Cherry expression on D20 (first day of S6) and D40 (end of S6) in SC islets. Scale bar, 200 µm. **b**,**c**, Proinsulin (PROINS) (**b**) and insulin (INS) (**c**) content determined by ELISA and normalized to the count of C-PEP^+^ cells determined by flow cytometry (in **b**, *n* = 4; in **c**, *n* = 5; mean ± s.d., unpaired two-tailed *t*-test). **d**, dGSIS at D40 normalized to total DNA content (*n* = 3, mean ± s.e.m.). **e**, Representative flow cytometry plots showing the lentiviral overexpression (OE) of *IIR-Venus* or *GFP*; SC islets were transduced on D19 and analysed on D24. **f**,**g**, Representative histogram (**f**) and quantification (**g**) of the flow cytometry analysis of the median inceptor intensity in the GFP^+^ or Venus^+^ population in inceptor–Venus or GFP control OE SC β cells (in **g**, *n* = 3; mean ± s.d., paired two-tailed *t*-test). **h**,**i**, Representative histogram (**h**) and quantification (**i**) of the flow cytometry analysis of the C-PEP–Cherry intensity in the C-PEP–Cherry^+^ population in inceptor–Venus or GFP control overexpressing SC β cells. (in **i**, *n* = 3; mean ± s.d., paired two-tailed *t*-test). **j**,**k**, Proinsulin (**j**) and insulin (**k**) content after a 6 h cycloheximide treatment (CHX, 100 µg ml^−1^) measured by ELISA and normalized to untreated samples (*n* = 5; mean ± s.d.; unpaired two-tailed *t*-test). **l**, Proinsulin content after a 6 h lysosomal inhibitor treatment (LI, pepstatin A, 10 μg ml^−1^ and E64d, 10 μg ml^−1^) measured by ELISA and normalized to untreated samples (*n* = 4; mean ± s.d.; unpaired two-tailed *t*-test). Source data

**Extended Data Fig. 5:**
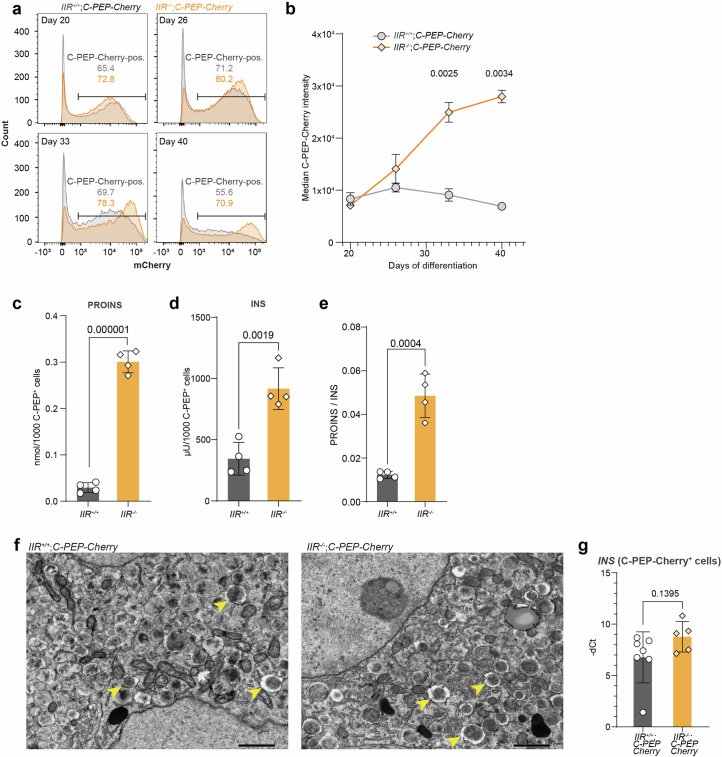
Inceptor regulates C-PEP-Cherry, insulin, and proinsulin content of SC β cells. **a, b**, Representative flow cytometry histograms (**a**) and quantification (**b**) of the C-PEP-Cherry intensity of the C-PEP-Cherry^+^ population of SC islets throughout S6 (**b**, *n* = 3, median ± s.d., two-way ANOVA followed by Šidák’s multiple comparisons test). **c, d**, Proinsulin (**c**) and insulin (**d**) content at D40 determined by ELISA and normalized to the count of C-PEP^+^ cells determined by flow cytometry (*n* = 4, mean ± s.d., unpaired two-tailed *t*-test). **e**, Ratio of proinsulin (**c**) to insulin (**d**) content measured by ELISA of SC islets on D40 (*n* = 4, mean ± s.d., unpaired two-tailed *t*-test). **f**, Epon embedding, ultrathin sectioning and TEM analysis of SC islets of D40; yellow arrows indicate mSGs Scale bar, 1 µm. **g**, qPCR analysis of INS in sorted C-PEP-Cherry^+^ cells; negative dCt values were calculated against HPRT (IIR^+/+^;C-PEP-Cherry, *n* = 7, IIR^-/-^;C-PEP-Cherry, *n* = 5, mean ± SD, *n* = 7, unpaired two-tailed *t*-test). Source data

In addition to increased β cell mass, *Iir*^−^^/^^−^ mice are characterized by an elevated pancreatic insulin content and fasting serum insulin levels at birth^29^. Therefore, we next measured insulin stores of *IIR*^−/−^; *C-PEP-Cherry* SC β cells. Strikingly, live analysis of C-PEP–Cherry by fluorescence microscopy and flow cytometry demonstrated greatly increased C-PEP–Cherry reporter activity in *IIR*^−^^/−^; *C-PEP-Cherry* SC β cells after prolonged S6 culture (Fig. 2a and Extended Data Fig. 5a,b). The increased C-PEP–Cherry reporter activity closely mirrored the proinsulin and insulin contents, which were significantly increased in *IIR*^−/−^; *C-PEP-Cherry* SC β cells (Fig. 2b,c). Given the increased differentiation efficiency and insulin content of the *IIR*^−/−^; *C-PEP-Cherry* SC β cells, we next investigated insulin secretion from *IIR*^−/−^; *C-PEP-Cherry* SC islets. Dynamic GSIS (dGSIS) was markedly improved in *IIR*^−/−^; *C-PEP-Cherry* SC islets compared to control SC islets, even surpassing 1^st^ phase (but not 2^nd^ phase) dGSIS of isolated human islets (Fig. 2d). To exclude clonal variation or effects by the C-PEP–Cherry reporter, we confirmed that insulin and proinsulin contents are also significantly higher in the *IIR*^−/−^ SC β cells than in *IIR*^+/+^ SC β cells, which do not contain the C-PEP–Cherry reporter (Extended Data Fig. 5c,d). However, the proinsulin to insulin ratio was significantly higher in *IIR*^−/−^ SC β cells than in *IIR*^+/+^ SC β cells, showing that the marked proinsulin increase did not result in an equimolar increase in insulin, possibly owing to insufficient conversion capacities of SC β cells (Extended Data Fig. 5e). Additionally, we established a lentiviral overexpression model in S6 SC islets. In agreement with our hypothesis, overexpression of inceptor–Venus (Fig. 2e–g) was sufficient to reduce the cellular content of C-PEP–Cherry compared to a GFP overexpression control (Fig. 2h,i). We investigated whether the increased insulin content changed proinsulin processing and granule formation. We analysed SG morphology by transmission electron microscopy (TEM) and found mSGs characterized by a dense core and halo in both *IIR*^−^^/−^; *C-PEP-Cherry* and *IIR*^+/+^; *C-PEP-Cherry* SC β cells (Extended Data Fig. 5f).

To determine the molecular mechanism of proinsulin accumulation in *IIR*^−/−^ SC islets, we measured proinsulin turnover at the stage of gene transcription and protein degradation. C-PEP–Cherry-sorted *IIR*^−/−^; *C-PEP-Cherry* SC β cells showed only a slight non-significant increase in *INS* gene transcription compared to control *IIR*^+/+^; *C-PEP-Cherry* SC β cells (Extended Data Fig. 5g). To determine proinsulin stability, we measured proinsulin content in *IIR*^+/+^; *C-PEP-Cherry* and *IIR*^−/−^; *C-PEP-Cherry* SC islets treated with cycloheximide (CHX), an inhibitor of translation. Notably, *IIR*^−/−^; *C-PEP-Cherry* SC islets retained more proinsulin during the 6 h CHX treatment (Fig. 2j). Insulin levels during the CHX treatment followed the same trend but were not significantly different between *IIR*^+/+^; *C-PEP-Cherry* and *IIR*^−/−^; *C-PEP-Cherry* SC islets (Fig. 2k). To investigate whether reduced lysosomal degradation is responsible for the retention of proinsulin in *IIR*^−/−^ SC β cells, we treated *IIR*^+/+^ and *IIR*^−/−^ SC islets with lysosomal inhibitors. During the 6 h treatment period, *IIR*^+/+^ but not *IIR*^−/−^ SC islets accumulated proinsulin (Fig. 2l). This finding indicates that inceptor contributes to proinsulin degradation under standard culture conditions with 8 mmol l^−1^ glucose and 2% bovine serum albumin.

### Inceptor is a TGN–endolysosomal trafficking receptor

**Fig. 3:**
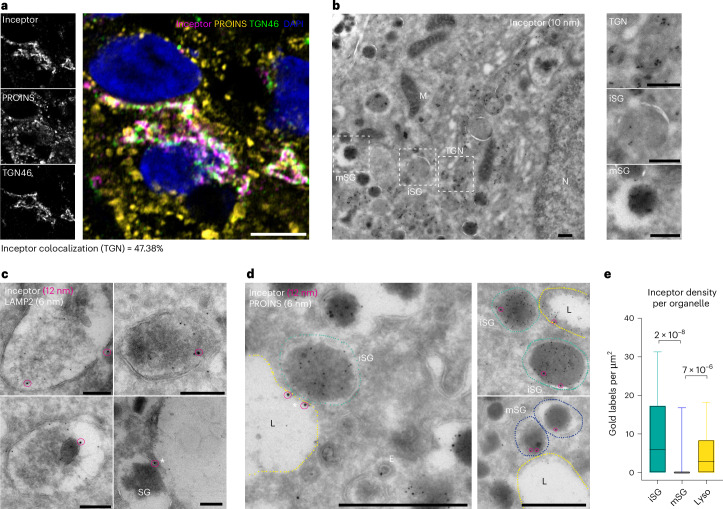
Inceptor localized to the TGN, SGs and lysosomes. **a**, Representative confocal image of *IIR*^+/+^ SC islets showing colocalization of inceptor with the TGN marker TGN46. Scale bar, 5 µm. Colocalization was quantified as the percentage of double-positive pixels and total inceptor pixels (*n* = 3, for each *n*, five or six images with approximately eight SC β cells each were analysed). **b**, Representative TEM overview image of immunogold staining of inceptor in human β cells. Scale bars, 250 nm. **c**, Representative images of lysosomes of human β cells stained for inceptor (12 nm gold particles, circled by magenta overlays) and LAMP2 (6 nm). Scale bars, 200 nm (*n* = 1). **d**,**e**, Representative images (**d**) and quantification (**e**) of human β cell organelles in islets immunostained for inceptor (12 nm diameter gold particles; circled by magenta overlays) and proinsulin (6 nm gold particles) (in **e**, *n* = 72, 395 and 91 organelles, respectively, Kruskal–Wallis test with Dunn’s multiple comparison post-hoc test. Centre line, median; box limits, upper and lower quartiles; whiskers, 10^th^–90^th^ percentile; outliers not displayed. Scale bars, 500 nm. Asterix marks inceptor-positive SG–lysosome fusion sites. N, nucleus; M, mitochondrion; E, endosomes. iSGs in cyan; mSGs in blue; lysosomes (L) in yellow. Source data

**Extended Data Fig. 6:**
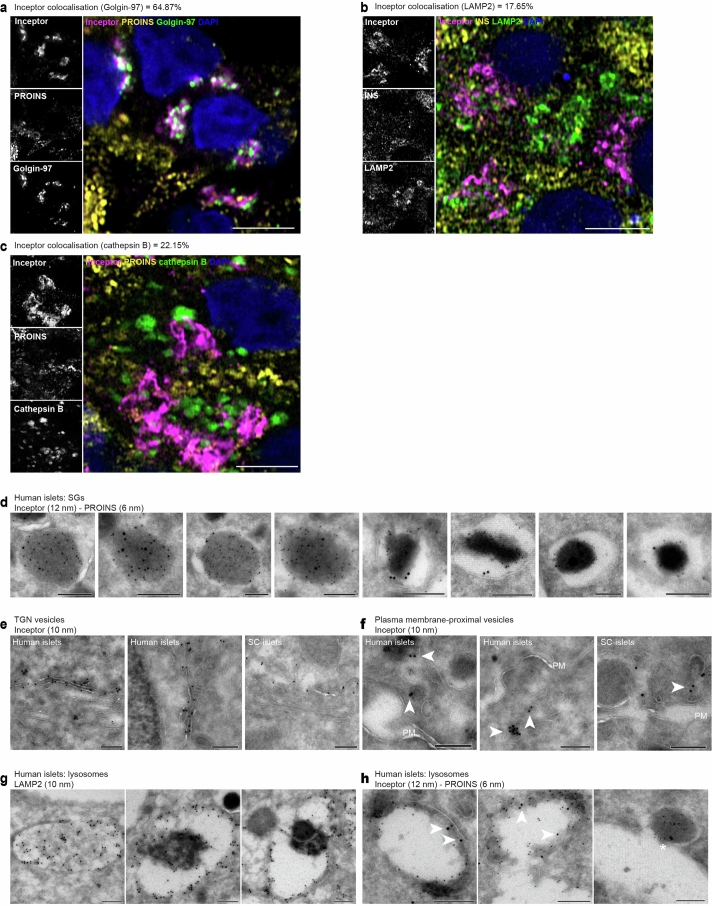
Analysis of the subcellular localization of inceptor in human primary β cells and SC β cells. **a-c**, Representative confocal images of IIR^+/+^ SC-islets, showing colocalization of inceptor with the TGN marker Golgin-97 (**a**), partial colocalization of inceptor with the lysosomal markers LAMP2 (**b**) and cathepsin B (**c**). Scale bars, 5 µm. Colocalization was quantified as the percentage of double-positive pixels and total inceptor pixels (*n* = 3, for each *n*, 5 to 6 images with approximately 8 SC β cells were analysed). **d-h**, Tokuyasu immunogold labelling of human primary islets or *IIR*^*+/+*^*;C-PEP-Cherry* SC-islets. **d**, Representative inceptor^+^ immature (four left panels) and mature (four right panels) SGs. **e, f**, Representative inceptor^+^ TGN (**e**) and plasma membrane-proximal (**f**) vesicles. **g**, Representative images showing the morphology of LAMP2^+^ lysosomes. **h**, Exemplary images showing proinsulin and inceptor-containing lysosomes. White arrows indicate inceptor staining and white asterisks mark inceptor at iSG-lysosome fusion sites. Scale bars, 200 nm, (*n* = 1) (**d-h**).

To understand the mechanism behind the reduced lysosomal degradation of proinsulin in *IIR*^−/−^; *C-PEP-Cherry* SC β cells, we investigated inceptor’s subcellular localization. Immunofluorescence in SC islets demonstrated that inceptor colocalizes with the TGN markers TGN46 and Golgin-97, and to a lesser extent with lysosomal markers LAMP2 and cathepsin B (Fig. 3a and Extended Data Fig. 6a–c). This subcellular localization pattern was supported by immunogold labelling and TEM of SC islets and isolated human islets, demonstrating that inceptor localized mainly to the sorting TGN compartment and, to a lesser extent, to SGs (Fig. 3b and Extended Data Fig. 6d,e), plasma-membrane-proximal vesicles (Extended Data Fig. 6f) and lysosomes (Fig. 3c and Extended Data Fig. 6g). By analysing the inceptor immunogold signal density by TEM, we found the inceptor signal to be more enriched in iSGs than in mSGs and that lysosomes contain low levels of the inceptor immunolabel (Fig. 3d,e). Intriguingly, we found inceptor to localize to iSG–lysosome fusion sites (Fig. 3c,d and Extended Data Fig. 6h, marked by asterisks).

**Fig. 4:**
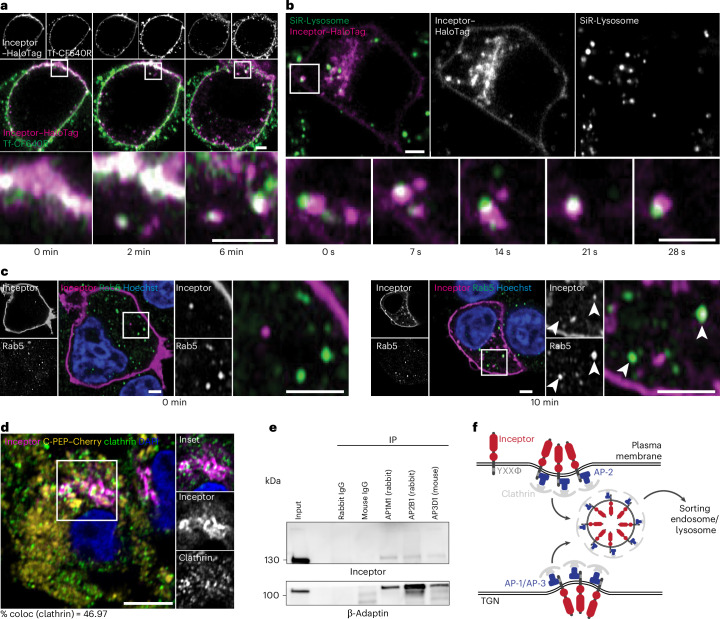
Inceptor is a TGN–endolysosomal trafficking receptor. **a**, Representative time points from live imaging of INS1-E cells, overexpressing inceptor–HaloTag, pulse-labelled with the AF488 HaloTag ligand and fluorescent transferrin (Tf-CF640R; time interval, 2 min) during cold-shock treatment, chased at 37 °C for the indicated duration. Scale bar, 2 µm (*n* = 3). **b**, Representative time points from live imaging of INS1-E cells, overexpressing inceptor–HaloTag, labelled with the AF488 HaloTag ligand and SiR-lysosome (time interval, 1 s). Scale bar, 2 µm (*n* = 3). **c**, Representative images of INS1-E cells, showing the internalization of inceptor–HaloTag pulse-labelled with a cell-impermeable AF488 HaloTag ligand during cold exposure and fixed after 0 min or 10 min of chase at 37 °C, then counterstained with anti-Rab5. Scale bar, 2 µm. White arrows, inceptor and Rab5 double-positive vesicles (*n* = 3). **d**, Representative confocal images of *IIR*^+/+^; *C-PEP-Cherry* SC islets showing partial colocalization of inceptor with clathrin. Scale bar, 5 µm. Colocalization was determined as the percentage of clathrin-inceptor double-positive pixels over all inceptor-positive pixels (*n* = 2; for each *n*, five or six images with approximately eight SC β cells were analysed). **e**, Representative co-immunoprecipitation of inceptor with AP-1, AP-2 and AP-3 in INS1 cells (*n* = 3). **f**, Schematic representation of inceptor in CCV formation at the plasma membrane and TGN.

We further investigated the trafficking patterns of inceptor by high-resolution live imaging in the rat insulinoma cell line INS-1E, overexpressing the inceptor–HaloTag fusion protein. After pulse-labelling with a cell-impermeable HaloTag ligand and fluorescently labelled transferrin during cold exposure, we chased the internalization of inceptor–HaloTag from the plasma membrane. Inceptor–HaloTag partially internalized in transferrin-positive vesicles, suggesting that its internalization follows a clathrin-dependent pathway (Fig. 4a). When co-stained with the fluorogenic SiR-lysosome dye, we observed that inceptor–HaloTag-positive vesicles co-migrate with lysosomes (Fig. 4b). Additionally, in fixed and post-stained cells, we found inceptor–HaloTag in Rab5-positive early endosomes shortly after internalization (Fig. 4c). By immunofluorescence imaging, we found inceptor colocalizing with clathrin-positive vesicles in SC β cell sections in the perinuclear and TGN regions (Fig. 4d). This demonstrates that inceptor is a TGN–endosomal and lysosomal trafficking receptor.

Inceptor, like the M6PRs, is internalized from the plasma membrane by the adaptor protein AP-2 (ref. ^29^). CI-M6PR and CD-M6PR are bound by AP-1, which mediates intracellular budding of CCVs from the TGN, which are trafficked towards sorting endosomes and lysosomes. Mechanistically, we found AP-1, AP-2 and AP-3 co-immunoprecipitated with inceptor in the rat insulinoma cell line INS-1 (Fig. 4e).

Taken together, these results suggest that the adaptor proteins mediate intracellular vesicular transport of inceptor between the TGN, endosomal–lysosomal system and plasma membrane, as described for CI-M6PR and CD-M6PR (Fig. 4f)^35^.

### Inceptor binds insulin and proinsulin

**Fig. 5:**
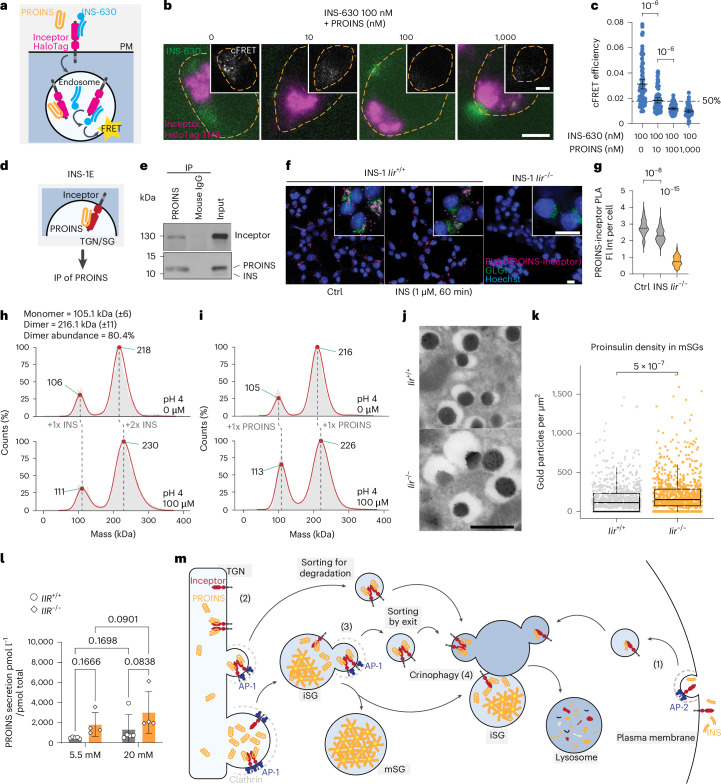
Insulin and proinsulin are inceptor binding partners. **a**, Schematic overview of FRET between overexpressed inceptor–HaloTag labelled by tetramethylrhodamine (TMR) and fluorescently tagged INS-630. PM, plasma membrane. **b**,**c**, Representative images (**b**) and quantifications of efficiency (**c**) of FRET between inceptor–HaloTag labelled with TMR and INS-630 in INS1-E cells co-treated with 100 nM INS-630 and the indicated amounts of proinsulin; the resulting corrected FRET (cFRET) signal is shown in the sub-panels (in **b**, scale bar, 5 µm, the orange dashed lines indicate approximate cell boundaries, inceptor is shown in purple, localizing to the Golgi region and labelled insulin, in green, accumulates at the cell periphery; in **c**, *n* = 81, 72, 67 and 50 cells, respectively, mean ± 95% CI, Brown–Forsythe and Welch one-way ANOVA followed by Dunnett’s T3 multiple comparisons test; 50% corresponds to half of the maximum signal). **d**, Schematic overview of the analysis of the binding of inceptor and endogenous proinsulin in INS1-E cells. **e**, Representative blot of the co-immunoprecipitation of endogenous inceptor with endogenous proinsulin (*n* = 3). **f**,**g**, Representative images (**f**) and quantification (**g**) of PLAs between endogenous inceptor and proinsulin, performed on INS-1 *Iir*^+/+^ or *Iir*^−/−^, treated with vehicle or insulin and counterstained with the Golgi marker GLG1; the counterstain is only shown in the magnified sub-panels to avoid obscuring the PLA signal in the full fields (in **f**, scale bar, 10 µm; in **g**, *n* = 196, 239 and 208 cells, respectively, median, Kruskal–Wallis one-way ANOVA followed by Dunn’s multiple comparisons test). **h**,**i**, Inceptor binding of insulin (**h**) or proinsulin (**i**) determined by CDMS. **j**,**k**, Representative TEM images (**j**) and quantification (**k**) of the density of proinsulin immunogold labelling in mSGs in pancreatic sections from *Iir*^+/+^ and *Iir*^−/^^−^ mice (in **k**, Wilcoxon two-tailed rank test with continuity correction; *n* = 775 mSG (wild-type) and 752 mSG (KO) from eight cells each; each dot in the plot represents an mSG; centre line, median; box limits, upper and lower quartiles; whiskers, 1.5 times the interquartile range; scale bar, 500 nm). **l**, Secreted PROINS normalized to total PROINS content of *IIR*^+/+^ or *IIR*^−/−^ SC islets in fully supplemented medium with 5.5 mM or 20 mM glucose after 24 h culture (*n* = 5 for *IIR*^+/+^ and *n* = 4 for *IIR*^−/−^; mean ± s.d., repeated-measures two-way ANOVA). **m**, Schematic summary of inceptor-mediated regulation of insulin homeostasis. At the plasma membrane, inceptor is internalized together with insulin and binds it in a pH-dependent manner (1). At the TGN and in maturing granules (2), inceptor binds proinsulin and induces its removal towards lysosomes (3) in a combination of sorting by exit and by degradation. Inceptor is present at the maturing granule–lysosome fusion site, where it might promote crinophagy or degradation of granules that fail the maturation process (4). AP-1/2, adaptor protein 1/2. Source data

**Extended Data Fig. 7:**
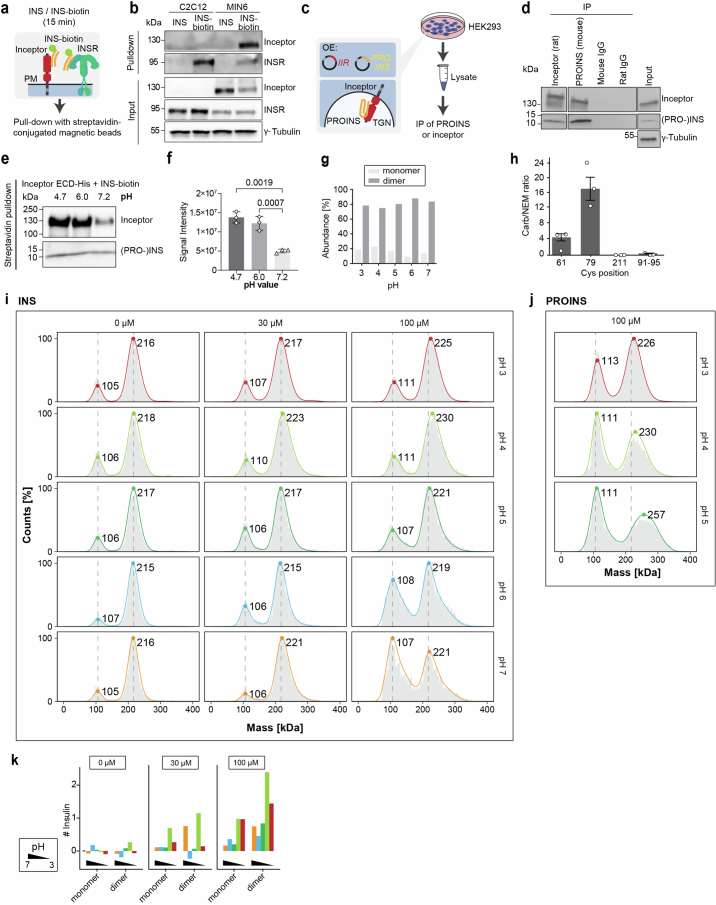
pH-dependent binding of inceptor to insulin and proinsulin. **a**, Schematic overview of the analysis of the binding of inceptor or INSR and exogenous biotinylated insulin (INS-biotin) with inceptor. **b**, Representative blot of the streptavidin pull-down of inceptor and the INSR with INS-biotin in inceptor-expressing MIN6 and inceptor-negative C2C12 cells. (*n* = 3). **c**, Experimental setup to analyse the binding of inceptor and proinsulin in HEK293 cells overexpressing C-terminally tagged Inceptor-HaloTag and human preproinsulin. **d**, Representative blot of the co-IP of inceptor with proinsulin and *vice versa* (*n* = 3). **e, f**, Representative blot (**e**) and quantification (**f**) of the streptavidin pulldown of inceptor-ectodomain (ECD)-His and insulin (**f**, *n* = 3; mean ± s.d.; one-way ANOVA followed by Šidák’s multiple comparisons test). **g**, Ratio of dimer vs. monomer determined by CDMS at various pHs. **h**, Disulfide bond determination by differential alkylation, expressed as ratios where higher is more likely to be involved in a disulfide bridge (*n* = 3, mean ± s.d.). **i, j**, Binding of insulin (**i**) or proinsulin (**j**) at different concentrations and pH to inceptor (monomer 105 kDa, or dimer 216 kDa) as determined by CDMS. **k**, Quantification of the number of insulin molecules bound to inceptor monomer or dimer under various pH levels and insulin concentrations. Source data

Considering the structural similarities between the cysteine-rich domains of inceptor and the INSR^29^, we reasoned that inceptor directly binds insulin and/or proinsulin to regulate the internalization of exogenous insulin or trafficking of newly synthesized proinsulin. To investigate whether inceptor binds insulin, we added insulin–biotin to the mouse insulinoma MIN6 cells and observed co-precipitation of inceptor with insulin–biotin (Extended Data Fig. 7a,b). We further performed fluorescence resonance energy transfer (FRET) experiments in inceptor–HaloTag overexpressing INS-1E cells incubated with fluorescent insulin–bodipy 630/650 (INS-630) (Fig. 5a). We observed a cytosolic FRET signal between inceptor–HaloTag and INS-630, especially localized in the inceptor-bright regions that correspond to the Golgi structures (Fig. 5b). Intriguingly, the addition of unlabelled proinsulin successfully reduced FRET between inceptor–HaloTag and INS-630 in a dose-dependent manner (Fig. 5c). Proinsulin and insulin competition at a 1:10 molar ratio resulted in a 50% reduction of the FRET signal of inceptor to INS-630, showing that inceptor binding to insulin can be efficiently displaced by proinsulin, even at low molar ratios (Fig. 5c).

Next, we examined the ability of inceptor to bind proinsulin. Inceptor and proinsulin co-immunoprecipitated in HEK293 cells overexpressing proinsulin and inceptor (Extended Data Fig. 7c,d). Likewise, the interaction between endogenous inceptor and proinsulin was detected by proinsulin immunoprecipitation in INS-1E cells (Fig. 5d,e). We also observed close proximity (<40 nm) of endogenous proinsulin and inceptor in INS-1 cells by proximity ligation assay (PLA), mostly found in the Golgi protein 1 (GLG1)-positive area (Fig. 5f). The PLA signal could be reduced by the addition of exogenous insulin to live cells (Fig. 5f,g). These findings together show that inceptor binds insulin and proinsulin across different cell lines.

As endosomal vesicles and SGs acidify in their maturation process, we wondered whether the inceptor–insulin interaction is pH-dependent. Interestingly, insulin–biotin pulldowns of the purified inceptor ectodomain revealed an increased inceptor–insulin binding at acidic pH (Extended Data Fig. 7e,f). Next, we obtained quantitative results by charge detection mass spectrometry (CDMS). Inceptor prevalently forms dimers, which are stable across a wide pH range (Extended Data Fig. 7g). Using differential alkylation, we discovered that Cys79 and, less prominently, Cys61 are engaged in disulphide bridges (Extended Data Fig. 7h). This suggests that they could be involved in inceptor dimerization. At acidic pH, the inceptor monomer binds up to one insulin molecule and the inceptor dimer can bind up to two insulin molecules (Fig. 5h). Both the inceptor monomer and dimer can bind one proinsulin molecule (Fig. 5i). We additionally show the pH-dependent and concentration-dependent binding of insulin to inceptor and observed that insulin bound to inceptor most readily at pH 4 (Extended Data Fig. 7i–k).

### Inceptor removes proinsulin from the secretory pathway

To further investigate the proinsulin binding and trafficking mediated by inceptor, we analysed the proinsulin content of mSGs in murine β cells. *Iir*^−/−^ murine pancreatic sections revealed a higher quantity of proinsulin present in mSGs by immunogold labelling than in wild-type controls (Fig. 5j,k). Consequently, we investigated proinsulin secretion in SC islets. *IIR*^−/−^ SC islets tended to secrete more of their proinsulin content over a 24 h culture period in 20 mmol l^−1^ glucose than did *IIR*^+/+^ SC islets (Fig. 5l). These results together suggest that in the absence of inceptor, there is a mild increase in proinsulin present in mSGs. Therefore, the physiological role of inceptor might be the prevention of proinsulin secretion by the secretory pathway; however, we cannot rule out an additional role of inceptor in proinsulin processing.

In summary, inceptor regulates insulin and proinsulin homeostasis in SC β cells. At the plasma membrane, inceptor is internalized by CCVs together with INSR, IGF1R and insulin. At the TGN or iSG, inceptor binds proinsulin and mediates its trafficking by CCVs towards lysosomal degradation in a mechanism involving both ‘sorting by exit’ and ‘sorting for degradation’. Moreover, inceptor can be found at sites of granule–lysosome fusion, suggesting its potential involvement in crinophagy (Fig. 5m).

### Targeting inceptor with mABs increases insulin stores

**Fig. 6:**
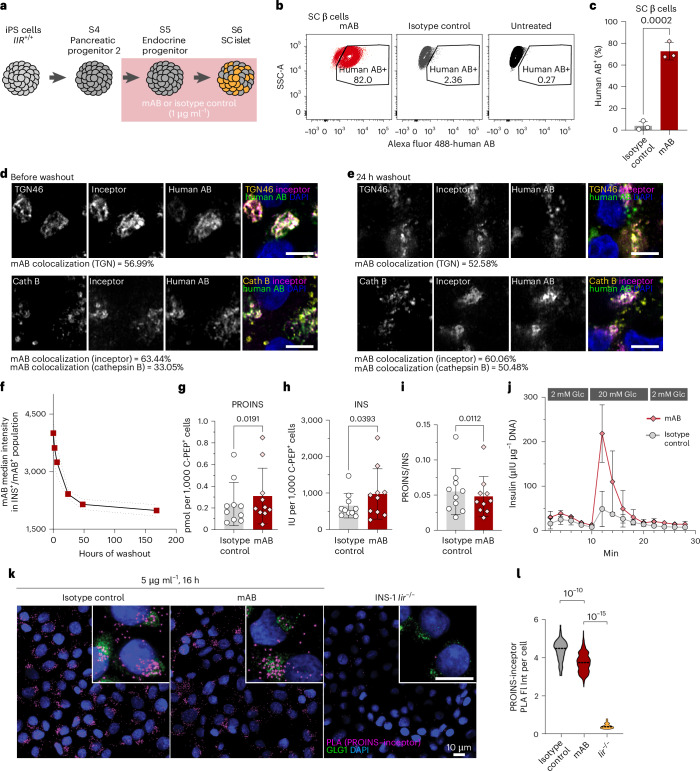
Targeting inceptor with mABs leads to increased insulin stores and secretion of SC β cells. **a**, SC islets were treated during S5 and S6 with an inceptor-specific humanized mAB or isotype control. **b**,**c**, Representative flow cytometry plots of mAB internalization in SC β cells (**b**) and quantification (**c**) (*n* = 3, mean ± s.d., unpaired two-tailed *t*-test). **d**,**e**, Subcellular localization of the internalized mAB in SC β cells before (**d**) or after (**e**) a 24 h washout period as determined by immunostaining for human mAB, inceptor, the TGN marker TGN46 and the lysosomal marker cathepsin B (Cath B). Scale bar, 5 µm. Colocalization was quantified as the percentage of double-positive pixels and total inceptor pixels (*n* = 5 (TGN), *n* = 6 (inceptor and cathepsin B) images from the same experiment with approximately eight SC β cells each). **f**, Flow cytometry quantification of mAB staining intensity of INS^+^ SC islet cells during mAB washout (*n* = 4; mean ± s.d.). **g**–**i**, Proinsulin (**g**) and insulin (**h**) content or proinsulin to insulin molar ratio (**i**) of SC islets analysed by ELISA and normalized to the count of C-PEP^+^ SC β cells determined by flow cytometry (*n* = 10, mean ± s.d., paired two-tailed *t*-test) **j**, dGSIS of mAB-treated or isotype-control-treated SC islets normalized to the total DNA content (*n* = 3, mean ± s.e.m.). **k**,**l**, Representative images (**k**) and quantification (**l**) of PLA of endogenous inceptor and proinsulin, performed on INS-1 *Iir*^+/+^ treated with anti-inceptor mAB or isotype control or INS-1 *Iir*^−/−^ and counterstained with the Golgi marker GLG1; the counterstain is shown only in the magnified sub-panels to avoid obscuring the PLA signal in the full fields (in **k**, scale bars, 10 µm; in **l**, *n* = 151, 295 and 150 cells, respectively; median, Kruskal–Wallis one-way ANOVA followed by Dunn’s multiple comparisons test). Source data

Finally, we aimed to modify proinsulin and insulin stores of genetically unaltered SC islets with mABs targeting the extracellular ligand-binding domain of inceptor throughout S5 and S6 (Fig. 6a). Over 60% of SC β cells internalized the inceptor mAB but not the control antibody (Fig. 6b,c). The internalized antibody colocalized with endogenous inceptor and showed similar colocalization with the TGN and lysosomes (Fig. 6d). To investigate the stability and action of the mAB, we performed a 24 h washout and found that the inceptor mAB still colocalizes with the TGN, inceptor and lysosomes (Fig. 6e); the mAB intensity decreased by around 50% within this washout period (Fig. 6f). This suggests that internalized mAB partially remains bound to inceptor throughout its cycling from the plasma membrane to the endosomal–lysosomal system and TGN. We then tested whether the mAB treatment recapitulates the phenotype of *IIR*^−/−^ SC islets. Interestingly, we found an increase in both the proinsulin and insulin content of SC β cells treated with the mAB compared to the isotype control (Fig. 6g,h). The molar ratio of intracellular proinsulin to insulin was only slightly decreased (Fig. 6i), probably because of the less pronounced proinsulin increase compared to inceptor KO SC islets (Fig. 2b). Importantly, the dGSIS of mAB-treated SC islets was markedly improved compared to isotype-control-treated islets (Fig. 6j). We reasoned that the antibody-bound inceptor might not be available for proinsulin binding; therefore, we performed a PLA on mAB-treated or control-treated INS-1 cells. Treatment with inceptor mAB reduced the occurrences of the proximity of endogenous inceptor and proinsulin compared to control-treated INS-1 cells, suggesting that mABs can interfere with the inceptor–proinsulin interaction (Fig. 6k,l).

## Discussion

Recently, we described inceptor as a desensitizer of INSR and IGF1R signalling at the plasma membrane of murine β cells. We showed that inceptor internalizes together with INSR and IGF1R at the plasma membrane via AP-2 in CCVs and shields β cells from constitutive autocrine and paracrine insulin signalling^29,36^. Embryonic pancreases from *Iir*^–/–^ mice had an increase in β cell mass and proliferation. At the same time, newborn *Iir*^–/–^ mice showed increased insulin content in the serum and pancreas.

Here, we translated our previous findings to human β cells and demonstrate that the lack of inceptor increases human SC β cell differentiation, survival and function, similarly to our previous findings in murine β cells^29^. Simultaneously, we discovered that *IIR*^–/^^–^ SC β cells and SC β cells treated with inceptor-specific mABs have increased proinsulin and insulin content and GSIS. The increased proinsulin content of *IIR*^–/–^ SC β cells could be attributed to a lower rate of lysosomal degradation. A thorough characterization of the subcellular localization of inceptor demonstrated that inceptor was localized mainly intracellularly within the TGN–endolysosomal system including SGs and, to a lesser extent, in plasma-membrane-proximal vesicles. Analogously to the previously described role of inceptor at the plasma membrane, we found that the adaptor proteins AP-1–AP-3 are responsible for the intracellular trafficking of inceptor in CCVs. Furthermore, we identified proinsulin and insulin as ligands for inceptor.

Taken together, our findings suggest an intracellular role of inceptor as a lysosomal degradation receptor for proinsulin. It was previously speculated that membrane-bound receptors in CCVs could sort proinsulin into SGs (‘sort by entry’)^1^. Surprisingly, inceptor’s intracellular role at the TGN and extended TGN (iSGs) is to remove unprocessed proinsulin to control granule content. Specifically, inceptor retrieves proinsulin from vesicles of the regulated secretory pathway (‘sorting by exit’), in line with the function of M6PRs in SGs^8,37^, and mediates the fusion of vesicles of the regulated secretory pathway and lysosomes (‘sorting for degradation’).

Previous studies have indicated that inceptor regulates the degradation of long-lived proteins and is involved in autophagy^26^. Given its dominant localization to the TGN–granule interphase, we and others have suggested that inceptor has a granule-formation role across various tissues, including zymogen granule maturation in the stomach and acrosome formation in early spermatids^27,28^.

Limitations of this study and further avenues of research involve the fine molecular details of inceptor function in β cells. It is currently not clear if and at which stage of internalization inceptor binds to extracellular insulin. However, the higher affinity of inceptor to insulin with dropping pH suggests that a potential binding might take place in acidified endosomes. It is also currently unclear whether inceptor directly or indirectly regulates proinsulin conversion to insulin.

Intriguingly, a coding single-nucleotide polymorphism in ELAPOR1/inceptor has recently been found to associate with decreased fasting proinsulin plasma levels^38^, highlighting the translational relevance of our findings. Taken together, inceptor is a potential molecular target for insulin sensitization and replenishing insulin stores in human β cells. Our in vitro results also suggest that blocking inceptor with specific mABs has the potential to increase insulin stores and secretion in human β cells.

## Methods

### SC-line generation

The Ethics Committee of the Technical University Munich positively voted on non-commercial research on human iPS cells (219/20 S, 290/20 S). The human iPS cell line HMGUi001-A^33^ (here labelled *IIR*^+/+^) has been used to generate the SC lines analysed. Both the *IIR*^+/+^ line and its derivative HMGUi001-A-8 (ref. ^34^) with a *C-PEP-Cherry* knock-in (here labelled *IIR*^+/+^; *C-PEP-Cherry*) were targeted by CRISPR–Cas9 to generate the respective *IIR*^−/−^ lines according to a previously published protocol^39^. In brief, iPS cells were transfected with a targeting vector (pU6-(BbsI)-sgRNAs-CAG-Cas9-Venus-bpA; Addgene no. 86986) encoding four single-guide RNAs (sgRNAs) (Supplementary Table 1) with Lipofectamine Stem Transfection Reagent (Invitrogen) according to the manufacturer’s instructions. Individual clones derived from single transfected, Venus^+^ cells were genotyped by a forward (fwd) primer (ATGGCGACCCGCAGGTGAGC) and a reverse (rev) primer (CTGGCCTCCTCATGACGCCAGAC). A homozygous 50 bp deletion in exon 1 was confirmed by Sanger sequencing from the U6 promoter (Eurofins). Furthermore, karyotyping was performed on colcemid-treated cells in the logarithmic growth phase. A minimum of 20 metaphases per cell line were analysed by the standard G banding technique followed by categorizing using the International System for Human Cytogenic Nomenclature.

### iPS cell maintenance and differentiation

iPS cells were maintained in StemMACS iPS-Brew XF culture medium (Miltenyi Biotec) under standard culture conditions (37 °C, 5% CO_2_ and 95% humidity) and on Geltrex (Gibco) or Matrigel (Corning)-coated dishes. Cultures were regularly checked for mycoplasma contamination with the MycoAlert PLUS Mycoplasma Detection Kit (Lonza) according to the manufacturer’s instructions. Planar iPS cell cultures were split by detaching the cells with 0.5 mmol l^−1^ EDTA in PBS and seeding them in StemMACS iPS-Brew XF and Y-27632 (10 µM; SantaCruz), which was changed to StemMACS iPS-Brew XF without Y-27632 after 24 h. To generate aggregate suspension cultures, iPS cells were detached with Accutase (Sigma-Aldrich) and seeded in StemMACS iPS-Brew XF supplemented with Y-27632 to a 30 ml spinner flask (Reprocell) (0.6 to 0.8 × 10^6^ cells per ml), which was placed on an incubator-based magnetic stirrer (Cultistir, Able) set to 60 rpm. The aggregates were split every 3–4 days by dissociating in Accutase and seeding into StemMACS iPS-Brew XF supplemented with Y-27632. Then, 48 h after seeding, the medium was changed to StemMACS iPS-Brew XF without Y-27632. The iPS cell lines were differentiated towards SC islets with a six-stage protocol as previously described (Supplementary Tables 2 and 3)^32^. At the beginning of S6 (day 19), cells were reseeded to six-well ultra-low binding plates (Corning) at a density of 1 × 10^6^ cells per ml. Plates were placed on an incubator-based rotational shaker (MaxQ 2000 CO_2_ Plus; Thermo Fischer Scientific) set to 100 rpm. Experiments were carried out after 3 weeks of S6 culture (day 40). For the analysis of SC islets after long-term S6 culture, SC islets were cultured until day 61. Morphology and C-PEP–Cherry expression were monitored with an EVOS M5000 microscope (Invitrogen).

### Human donors

#### Human islets

Human islets for research were provided by the Alberta Diabetes Institute IsletCore at the University of Alberta in Edmonton, Alberta, Canada (www.bcell.org/adi-isletcore), with the assistance of the Human Organ Procurement and Exchange (HOPE) programme, Trillium Gift of Life Network (TGLN) and other Canadian organ procurement organizations. Islet isolation was approved by the Human Research Ethics Board at the University of Alberta (Pro00013094). All donors’ families gave informed consent for the use of pancreatic tissue in research. The Ethics Committee of the Technical University Munich positively voted on research on human material (557/16 S). The batches used and donor characteristics are summarized in Supplementary Table 4. Islets were derived from two female and two male donors. Donors had a body mass index of between 23.5 and 35.8. The HbA1c was unknown for one donor and ranged from 3.8 to 5.8 for the other three donors.

#### Human pancreatic tissue

Human foetal pancreatic tissues (11–12 weeks post-conception) were provided by the INSERM HuDeCA Biobank. Maternal written consent was obtained, along with approval from the French agency for biomedical research (Saint-Denis La Plaine, France). Human adult pancreases were removed from adult brain-dead organ donors before cardiac death with prior consent for research use at the Hôpital Saint-Louis (Paris, France)^40^. Human pancreatic tissue was processed in accordance with the French bioethics legislation and INSERM guidelines (French bylaw, published 29 December 1998).

### Molecular cloning

The *IIR-HaloTag* N-terminal fusion and *IIR-HaloTag* or *IIR-Venus* C-terminal fusion plasmids were obtained from Genescript as pGenlenti backbone inserts (NCBI reference sequence NM_020775.5). The HaloTag sequence was inserted in the *IIR* open reading frame between amino acids 43 and 44 for N-terminal tagging. The inserted sequence was flanked at the 5′ end by an EcoRI cutting site, a stop codon and a Kozak sequence, and at the 3′ end by a NheI cutting site. Silent mutations were introduced at amino acids P527 (TTC to TTT) and N815 (AAT to AAC). For C-terminal tagging, the HaloTag was inserted at the 3′ end of the *IIR* open reading frame. The resulting HaloTag constructs were subcloned into a pCAG vector (Addgene vector no. 79009 modified with an SV40 ori /pA) by EcoRI and NheI digestion.

The pLenti *IIR-Venus* construct (C-terminally tagged) was synthesized as described above and subcloned into a pLenti6 backbone^41^ by BamHI restriction.

### Immunostaining and image analysis

Differentiating aggregates were fixed with 4% PFA, dehydrated in a sucrose gradient (10–30% in PBS), frozen in tissue freezing medium (Leica) and cut into 15 µm-thick sections. Sections were rehydrated in PBS, permeabilized with 0.1% Triton-X-100 and blocked with blocking solution (10% FBS, 0.1% BSA, 3% donkey serum and 0.1% Tween-20 in PBS). Primary antibodies were diluted in blocking solution and incubated with the sections at 4 °C overnight. Slides were washed with PBS, incubated with secondary antibodies (1:500) for 4 h and stained with 2 µg ml^−1^ DAPI in PBS for 30 min. All antibodies are summarized in Supplementary Tables 5 and 6. Slides were mounted in Elvanol (25% glycerol, 10% Mowiol, 2% DABCO, 100 mmol l^−1^ Tris-HCl pH 8.0) and imaged at the Zeiss LSM 880 confocal microscope with or without Airyscan FAST. Image quantification and processing were performed with Zen Blue (v.3.9; Zeiss) or Fiji (ImageJ v.1.53o)^42^. Colocalization was determined with the Zen Blue (v.3.9) software, and the ‘colocalization coefficient’ expressed in percentage was used. For each multi-Z stack image, the values from images (numbers indicated in figure legends) were averaged and denoted below the corresponding panel.

Human foetal pancreatic sections (5 μm thickness) were prepared and processed from paraffin-embedded tissues as previously described^40^. Nuclei staining was performed with Hoechst 33342 (0.3 mg ml^−1^, Invitrogen). Images were acquired using a Leica DM 4000B microscope running Wasabi software (v.1.5) (Hamamatsu Photonics). The images were processed using Fiji^42^.

### Cell culture

MIN6 K8 cells, a gift from Jun-ichi Miyazaki, were cultured in high-glucose DMEM supplemented with 10% FBS, 50 µmol l^−1^ β-mercaptoethanol and penicillin (100 units per ml)–streptomycin (100 µg ml^−1^). INS-1 (C0018007, AddexBio) and INS-1E (C0018009, AddexBio) cells were cultured in RPMI-1640 (Gibco) supplemented with 10% FBS, 50 µmol l^−1^ β-mercaptoethanol, 1 mmol l^−1^ sodium pyruvate, 10 mmol l^−1^ HEPES, 1 mmol l^−1^ glutamine and penicillin–streptomycin. HEK293 (ATCC CRL-1573) and C2C12 (ATCC CRL-1772) cells were maintained in high-glucose DMEM supplemented with 10% FBS and penicillin–streptomycin.

### INS-1 *Iir*^−/−^ generation

INS-1 cells (AddexBio, C0018007) were targeted by CRISPR–Cas9 to generate the INS-1 *Iir*^−/^^−^ cell line according to a previously published protocol^39^. In brief, INS-1 cells were transfected with two targeting vectors (pU6-(BbsI)-sgRNAs-CAG-Cas9-Venus-bpA; Addgene no. 86986) encoding for sgRNAs directed against exon 2 (GTGACAGCACAGGTTCCAGG) and exon 3 (GTCCTGCAAGCCGTGTGCGG) with Lipofectamine 2000 (Invitrogen) according to the manufacturer’s instructions. Individual clones derived from single transfected cells were genotyped by three primers (TGTTGATGTTTGATCTCTGATGATGGACCG, CTCCCCAAGTGCTGGGATTAAGGT and GAAGTGCAGTTCTCGGTGGACTCT). The KO was confirmed by the lack of protein expression in immunofluorescence and western blot.

### FRET experiments

INS-1E cells were transfected using Lipofectamine 3000 (Thermo Fisher, L3000008) according to the manufacturer’s instructions with a plasmid encoding the inceptor–HaloTag fusion protein. After 48 h, the cells were incubated with the HaloTag ligand tetramethylrhodamine (Promega, G8251; 1:5,000) for 20 min. After extensive washing steps, 100 nmol l^−1^ of INS-630 and 0, 10, 100 and 1,000 nmol l^−1^ proinsulin were added to the medium for 40 min before imaging with a Zeiss LSM 880. To calculate the corrected FRET (cFRET) value, crosstalk was subtracted as previously described^43^; cFRET efficiency was calculated as the ratio of cFRET to donor fluorescence.

### Insulin–biotin and INS-630 synthesis

#### Insulin–azide

Human insulin (100 mg, 17.2 µmol) was dissolved in H_2_O and dimethylformamide (H_2_O/DMF) (2:1, 3.3 ml) and cooled to 0 °C under argon atmosphere. Triethylamine (48 µl, 17 µmol) was added to adjust the pH to 11. A solution of 2,5‑dioxopyrrolidin‑1‑yl 4‑azidobutanoate (5.45 mg, 24.1 µmol) in a mixture of DMF (496 µl) and 5% aqueous H_2_SO_4_ (4 µl) was added gradually in portions of 100 µl. Upon the start of the formation of disubstituted product, the reaction was stopped by adjusting the pH to 3 using 1 M aqueous HCl. The product was lyophilized, dissolved in a water and acetonitrile mixture (80:20, 3 ml) and purified by high-performance liquid chromatography (HPLC) using an automated Reveleris PREP Plant system (Buchi) on an RP-peptide column (Jupiter 10 µm Proteo 90 Å, LC column 250 ×30 mm; Phenomenex). This provided the B29-azide-modified insulin as a colourless lyophilizate (45 mg, 7.60 µmol, 45%).

#### INS-630

BDP630/650-alkyne (1.06 mg, 2.17 µmol) in DMF (100 µl) was added to a solution of azide-modified insulin (10.8 mg, 1.82 µmol) in deionized H_2_O (5 ml). A mixture of CuSO_4_ (1.14 mg, 4.56 µmol), Tris((1-hydroxy-propyl-1H-1,2,3-triazol-4-yl)methyl)amine (THPTA; 7.93 mg, 18.3 µmol), sodium ascorbate (7.23 mg, 36.5 µmol) and aminoguanidine hydrochloride (4.04 mg, 36.5 µmol) in H_2_O (433 µl) was added to the solution. The solution was allowed to stir for 27 h and the product was purified by HPLC (RP-peptide column Jupiter 4 μm Proteo 90 Å, LC column 250 ×10 mm; Phenomenex). After lyophilization, BDP630/650-labelled insulin (3.21 mg, 0.50 µmol, 28 %) was obtained as a bluish solid.

#### Insulin–alkyne

Human insulin (300 mg, 0.052 mmol) was dissolved in H_2_O/DMF (60 ml, 2:1) and cooled to 0 °C under argon atmosphere. Triethylamine (144 μl, 1.039 mmol) was added to the solution to adjust the pH to 10. A solution of hept-6-ynoic acid NHS ester (10.81 mg, 0.052 mmol) in a mixture of DMF (2.79 ml) and 5% aqueous H_2_SO_4_ (21 μl) was added gradually in portions of 300 μl. After observing the start of formation of the disubstituted product, the reaction was stopped by adjusting the pH of the reaction mixture to 3 using 1 M aqueous HCl. The reaction mixture was lyophilized. The lyophilizate was dissolved in a water and acetonitrile mixture (3 ml, 80:20) and purified by HPLC to yield B29-alkyne-modified insulin.

#### Biotin-PEG(9)-azide

To a solution of biotin *N*-hydroxysuccinimide ester (100 mg, 0.293 mmol) in DMF (2.5 ml), a PEG linker (NH_2_-PEG(9)-azide) (122 mg, 0.293 mmol) was added. The reaction was stirred for 44 h at 20–25 °C. The solvent was removed under reduced pressure and the residue was diluted in 1 ml dichloromethane (DCM). Upon adding diethyl ether, a colourless solid precipitated, which was filtered off, washed with diethyl ether (three times) and re-dissolved in DCM. DCM was removed under reduced pressure to produce a white solid (0.145 g, 0.218 mmol, 75%).

#### Insulin–biotin

A solution of biotin-PEG(9)-azide (8.43 mg, 12.7 μmol) in *tert*-butanol (100 μl) was added to a solution of alkyne-modified insulin (15 mg, 2.54 μmol) in deionized H_2_O (1 ml). A mixture of CuSO_4_ (1.58 mg, 6.34 μmol), THPTA (11.02 mg, 25.37 μmol), sodium ascorbate (10.05 mg, 50.74 μmol) and aminoguanidine hydrochloride (5.61 mg, 50.74 μmol) in H_2_O (603 μl) was added to the solution. The solution was allowed to stir for 41 h and the reaction mixture was purified using Amicon-15 centrifuge filter units (cutoff, 3 kDa). After lyophilization of the residue, the insulin–biotin (4.73 mg, 0.719 μmol, 29 %) was obtained as a white solid.

### Pulse-chase live imaging of cell surface inceptor

INS-1E cells were transfected using Lipofectamine 3000 with a plasmid encoding N-terminally tagged inceptor–HaloTag fusion protein. Then, 48 h after transfection, the cells were incubated on ice for 30 min with the cell-impermeable HaloTag ligand Alexa Fluor 488 (Promega, G1001; 1:1,000) and 50 μg ml^−1^ Human Transferrin CF640R (Biotium, BT00085). The ice-treated cells were released from the cold shock directly in the incubation chamber of a Zeiss LSM 880 (Airyscan FAST mode) and imaged or fixed at the indicated time points. Alternatively, transfected INS-1E cells were incubated for 30 min with the HaloTag ligand Alexa Fluor 488 and SiR-lysosome (Cytoskeleton CY-SC012, 1:1,000) at 37 °C and imaged after washing.

### PLA

INS-1 *Iir*^+/+^ or *Iir*^−/−^ cells were seeded into microwell plates (Ibidi) and treated the next day with 1 µmol l^−1^ insulin for 1 h or 1 µg ml^−1^ isotype control or mAB against the extracellular portion of inceptor overnight. The samples were processed according to the Duolink PLA Orange kit protocol (Sigma-Aldrich, DUO92102). The PLA reaction was performed between endogenous inceptor and proinsulin (Supplementary Table 5), followed by nuclear counterstain (Hoechst 33342; Thermo Fisher Scientific, 62249) and an immunofluorescence staining. Images were acquired using the Airyscan FAST mode on a Zeiss LSM 880, and segmentation was performed using Fiji to identify the nuclei, followed by Voronoi segmentation and quantification of PLA fluorescence intensity (thresholded with the Moments method) per Voronoi-identified cell.

### Flow cytometry

Differentiating aggregates were dissociated into single cells by Accutase. For live analysis of fluorescence, the cells were resuspended in PBS and analysed immediately. For fixation, the cells were resuspended in 4% PFA, washed with 5% FBS in PBS, permeabilized and blocked in FACS blocking solution (10% heat-inactivated FBS, 0.1% BSA, 3% donkey serum, 0.1% Tween-20 and 0.2% Triton-X-100 in PBS), incubated with primary antibodies at 4 °C overnight, washed with PBS, incubated with secondary antibodies (1:500) for 1 h at room temperature and washed with PBS. All antibodies were diluted in FACS blocking solution (Supplementary Tables 5 and 6). In negative controls, the primary antibody incubation was replaced by incubation in FACS blocking solution. For analysis, all cells were filtered and loaded onto a BD FACS Aria III flow cytometer. The selected subpopulations were analysed for their percentage or for median intensity by FlowJo (v.10.7.1).

### Perifusion assay for dGSIS

The flowthrough of 50–70 SC islets or isolated human islets incubated in different glucose concentrations was collected with the perifusion system PERI-4.2 (Biorep). The samples were pre-treated with Krebs-Ringer Buffer (KRB) (25.8 mmol l^−1^ NaCl, 0.96 mmol l^−1^ KCl, 0.24 mmol l^−1^ KH_2_PO_4_, 0.24 mmol l^−1^ MgSO_4_, 0.4 mmol l^−1^ CaCl_2_, 4.8 mmol l^−1^ NaHCO_3_, 10 mmol l^−1^ HEPES pH 7.4) supplemented with 0.1% BSA and 2 mmol l^−1^ glucose for 1 h and loaded into the perifusion system with a flow rate of 100 µl min^−1^. Flowthrough was collected in 2 min steps. The samples were perifused with KRB supplemented with 0.1% BSA and 2 mmol l^−1^ glucose during steps 1–4, 20 mmol l^−1^ glucose during steps 5–10 and 2 mmol l^−1^ glucose during steps 11–14. After the last step, the islets were collected and lysed in 0.2% SDS, 0.1 mol l^−1^ NaCl, 5 mmol l^−1^ EDTA, 0.1 mg ml^−1^ Tris-HCl pH 8.0 and 0.5 mg ml^−1^ proteinase K. DNA was precipitated with isopropanol and washed with ethanol. DNA concentration was measured by a Nanodrop 2000c (Thermo Fisher Scientific) and used for the normalization of insulin content in the flowthrough.

### Insulin and proinsulin ELISA

For proinsulin stability assays, SC islets were treated with CHX (100 µg ml^−1^; Merck) or lysosomal inhibitors pepstatin A (10 μg ml^−1^; Torcis) and E64d (10 μg ml^−1^; Torcis) or maintained in growth conditions for 6 h and processed as other untreated samples. The proinsulin secretion assay was performed with SC islets differentiated from SC-derived endocrine progenitors, cryopreserved at day 19 and recovered as previously described^44^. Cells were cultured for 2.5 weeks in standard S6 media and were then transferred in either 5.5 mM or 20 mM glucose containing S6 medium (Supplementary Table 3). After 24 hours, the proinsulin content of the supernatant was measured directly, and the cellular proinsulin content was measured as described below.

SC islets were dissociated into single cells with Accutase. Single cells were counted in a haemocytometer and part of the sample was used to determine the SC β cell percentage by flow cytometry. The remaining sample was sonicated in 50 µl water and lysed in 150 µl of 96% ethanol with 0.18 mol l^−1^ of HCl. Perifusion assay flowthrough samples, cell culture medium or cell lysates were analysed for insulin and proinsulin content by insulin and proinsulin ELISA (Mercodia 10-1113-01 and 10-1118-01) according to the manufacturer’s instructions. The measured concentrations were normalized to the total sample volume and the DNA content or cell count. For calculation of the proinsulin to insulin ratio, insulin content was converted from µU to nmol by multiplying by a conversion factor of 6.945.

### Lentiviral production and transduction

Human 293T (ATCC CRL-3216) cells were maintained in high-glucose DMEM (Gibco) supplemented with 10% FBS and geneticin (2 mg ml^−1^; Gibco). HEK293T cells cultured without antibiotics were co-transfected with pLenti-Puro6-inceptor-venus (generated as described above) or pLKO.1-puro-CMV-TurboGFP (Sigma-Aldrich), psPAX2 and pMD2.G (gift from Didier Trono; Addgene plasmid nos. 12260 and 12259) with Lipofectamine 3000 (Invitrogen) according to the manufacturer’s instructions with molar ratios 1:1:1; 16 h later, the medium was changed to Opti-MEM (Gibco) supplemented with glutaMAX (Gibco), 2% FBS and 1 mmol l^−1^ sodium pyruvate (Gibco). The supernatant was collected 48 h after transfection, filtered through a 45 µm filter. To transduce SC β cells, 500 µl of supernatant was added to the cell suspension in one well of a six-well ultra-low binding plate directly after the reaggregation step on day 19. After 5 days, SC islets were dissociated, fixed and analysed by flow cytometry.

### Animal experiments

Animal experiments were carried out in compliance with the German Animal Protection Act and with the approved guidelines of the Society of Laboratory Animals (GV-SOLAS) and the Federation of Laboratory Animal Science Associations (FELASA). The animals were housed under a 12:12 light–dark cycle at 22 ± 1 °C, 45–55% humidity and fed chow diet (Altromin 1314) ad libitum. Inceptor full-body KO mice were generated on a C57BL/6J background as described previously^28^. For TEM analysis of *Iir*^+/+^ and *Iir*^−/−^ murine islets, tissues of 4-month-old male and female mice were used.

### scRNA-seq

SC islets were dissociated by TripLE Select Enzyme (Gibco), and single cells, sorted by flow cytometry, were used for scRNA-seq library preparation with a target recovery of 10,000 cells. Libraries were prepared using the Chromium Next GEM Single Cell 3′ Kit (v.3.1) (10× Genomics, PN-1000121) according to the manufacturer’s instructions.

Libraries from *IIR*^−/−^; *C-PEP-Cherry* and *IIR*^+/+^; *C-PEP-Cherry* cell lines were pooled and sequenced according to 10× Genomics’ recommendations on an Illumina NovaSeq 6000 system with a target read depth of 50,000 reads per cell. Demultiplexed reads were aligned to the GRCh38 human genome and pre-processed using the CellRanger software (v.3.1.0) (10× Genomics) for downstream analyses. Ambient genes were estimated based on expression in empty droplets using DropletUtils^45^ (v.1.14.2). Genes with an ambient expression score larger than 0.0007 were considered ambiently expressed genes. To filter low-quality cells, droplets with fewer than 1,500 genes and with less than 3,500 or more than 40,000 unique molecular identifier counts were excluded. Cells with a mitochondrial count fraction smaller than 0.025 or greater than 0.4 were excluded. To obtain robust doublet estimates (expected doublet rate of 0.1), we used scrublet^46^ (v.0.2.3), DoubletDetection^47^ (v.4.2), scds^48^ (v.1.10.0), scDblFinder^49^ (v.1.11.4), DoubletFinder^50^ (v.2.0.3) and SOLO^51^ (implemented in scvi-tools v.0.17.1)^52^ to detect doublets and counted the number of times any cell was detected as a doublet. Cells consistently detected by four or more methods as a doublet were considered to be doublets and subsequently excluded from further analysis. Gene counts were normalized using sctransform^53^ (v.0.3.3) with v2 regularization and default parameters in Seurat (v.4.1.1)^54^. Corrected counts were used for subsequent analysis steps and visualizations unless stated otherwise. Samples were integrated using Harmony^55^ implemented in harmonypy (v.0.0.6). The latent spaces produced by Harmony were used for uniform manifold approximation and projection embeddings and Leiden clustering. To ensure optimal integration and embedding of the endocrine cell fraction, integration was repeated on only endocrine cells (that is, cells with high *CHGA* expression). Clustering and annotation were performed separately on endocrine (high *CHGA* expression) and non-endocrine cells (low *CHGA* expression), as with the integration. Final clusters were then annotated using known cell type marker genes (*INS*, *GCG*, *TPH1*, *SST*, *ARX*, *GAP43*, *MKI67*, *EPCAM*, *VIM*).

### Gene expression analysis by quantitative real-time PCR

SC islets were dissociated by Accutase on day 40, FAC-sorted for the C-PEP–Cherry signal and lysed in QIAzol lysis reagent (Qiagen). RNA was extracted by the RNeasy Micro Kit (Qiagen). For cDNA preparation, 50 ng RNA was processed by the SuperScript VILO cDNA Synthesis Kit (Invitrogen) according to the manufacturer’s instructions. The qPCR was performed on ViiA 7 Real-Time PCR System (Applied Biosystems), using the TaqMan Universal Master Mix II, no UNG (Applied Biosystems), with TaqMan primers for insulin Hs02741908_m1 and for HPRT Hs02800695_m1 (Thermo Fisher Scientific). Negative ΔCt values were calculated against HPRT.

### TEM

SC islets were dissociated by Accutase on day 33, and SC β cells with high C-PEP–Cherry were sorted and re-aggregated as described above. Murine pancreases were isolated from 4-month-old mice and dissected in 4% PFA. Re-aggregated SC β enriched SC islets, human islets or murine pancreases were fixed in 4% PFA in 100 mmol l^−1^ phosphate buffer (pH 7.4) and stored in 4% PFA or 1% PFA at 4 °C for Epon embedding into epoxy resin and for Tokuyasu cryosectioning, respectively.

For epoxy resin embedding, the samples were further processed according to a modified protocol for serial block face scanning electron microscopy^56^ using osmium tetroxide (OsO_4_), thiocarbohydrazide (TCH) and OsO_4_ again to generate enhanced membrane contrast^57,58^. In brief, samples were post-fixed overnight in modified Karnovsky fixative (2% glutaraldehyde and 2% formaldehyde in 50 mmol l^−1^ HEPES pH 7.4), post-fixated in a 2% aqueous OsO_4_ solution containing 1.5% potassium ferrocyanide and 2 mmol l^−1^ CaCl_2_ (30 min on ice), washed in water, 1% TCH in water, rinsed in water and incubated a second time in 2% OsO_4_. Samples were washed and en-bloc contrasted with 1% uranyl acetate (UA), washed in water and dehydrated in a graded series of ethanol in water, followed by three changes in pure ethanol on a molecular sieve. Samples were infiltrated into the Epon substitute EMBed 812 (resin in ethanol mixtures: 1:3, 1:1 and 3:1 for 1 h each, followed by pure resin overnight and for 5 h), embedded into flat embedding moulds and cured at 65 °C overnight. Ultrathin sections (70 nm) were prepared with a Leica UC6 ultramicrotome (Leica Microsystems) and a diamond knife (Diatome), collected on formvar-coated slot grids and then stained with lead citrate^59^ and UA.

For Tokuyasu cryosectioning and immunogold labelling, the samples were processed as previously described^58,60,61^. In brief, they were washed in phosphate buffer, infiltrated stepwise into 10% gelatine at 37 °C, cooled down on ice, cut into small blocks, incubated in 2.3 mol l^−1^ sucrose for 24 h at 4 °C, mounted on pins (Leica, no. 16701950) and plunge-frozen in liquid nitrogen. Then, 70–100 nm sections were cut on a Leica UC6 + FC6 cryo-ultramicrotome (Leica Microsystems) and picked up in methyl cellulose (MC) and sucrose (one part 2% MC, Sigma-Aldrich, M-6385, 25 cP; one part 2.3 mol l^−1^ sucrose) using a perfect loop. Ultrathin sections were stained with primary antibodies (Supplementary Table 5) for immunogold labelling^58,60^. Grids were washed with PBS, 0.1% glycine in PBS, blocked with 1% BSA in PBS and incubated with the primary antibodies for 1 h. For single labelling, the sections were washed in PBS and incubated with bridging antibodies^60^ (1:100), except for the proinsulin antibody, which was detected directly with protein A gold, followed by washes in PBS and incubation with protein A conjugated to 10 nm gold (1:25, UMC, Utrecht) for 1 h. For double labelling, the primary antibodies were applied simultaneously followed by washes in PBS and incubation with a combination of anti-rat 12 nm IgG gold anti-mouse 6 nm IgG gold in 1% BSA in PBS. Immunogold-labelled grids were washed in PBS, post-fixed in 1% glutaraldehyde (5 min), thoroughly washed in water to remove the PBS, contrasted with neutral uranyl oxalate (2% UA in 0.15 mol l^−1^ oxalic acid, pH 7.0) for 5 min, washed in water and incubated in MC containing 0.4% UA. Grids were looped out with a perfect loop and the MC–UA film was reduced to an even, thin film and air-dried. All sections were analysed on a JEM 1400Plus transmission electron microscope (JEOL) at 80 kV, and images were taken with a Ruby digital camera (JEOL).

### TEM image analysis

Immunogold-labelled inceptor and proinsulin densities in TEM images acquired from fixed human islets (24 cells) and mouse pancreatic sections of *Iir*^+/+^ (eight cells) and *Iir*^−/−^ (eight cells) animals were analysed using QuPath software (v.0.4.3)^62^. Immunogold particles were detected automatically by a trained pixel classifier. SGs were annotated using a pixel classifier and manual corrections. The density values were obtained by dividing the number of immunogold particles by the area of each organelle. SGs were divided into mSGs (low proinsulin levels, characterized by a halo) or iSG (highly proinsulin levels, enriched without a halo). The automatically detected immunogold labels were quantified in each annotated organelle. Data were analysed and plots were generated in R^63^ and GraphPad Prism (v.10.1.2).

### Co-immunoprecipitation, pulldowns and western blot

#### Proinsulin and inceptor co-immunoprecipitation

HEK293 cells were transfected with human proinsulin overexpressing plasmid (pTARGET-hProinsulin, gift from Peter Arvan^64^) or C-terminally tagged inceptor–HaloTag with Lipofectamine 3000 (Invitrogen) according to the manufacturer’s instructions. HEK293 or INS-1E cells were lysed in 2% NP-40, 1% Triton-X-100, 10% glycerol and 1% protease inhibitor cocktail (Sigma-Aldrich, P8340) in PBS for 30 min at 4 °C. The lysate was centrifuged for 10 min at 21,000*g* on a tabletop centrifuge and the supernatant was collected. Then, 20 μl SureBeads Protein G Magnetic Beads (Bio-Rad) were coupled to either an inceptor antibody and a corresponding isotype control (rat IgG2b) or a proinsulin antibody and a control mouse antibody (see Supplementary Table 5). The beads were washed with PBS-T and incubated in PBS-T with 2% BSA for 30 min then washed with lysis buffer. Next, 100 μg lysate (measured by Pierce BCA Protein Assay Kit; Thermo Fisher Scientific) was loaded onto the beads and incubated at 4 °C overnight. Then, the beads were thoroughly washed in PBS-T and the proteins were eluted in 2× Laemmli buffer at 100 °C for 5 min.

#### Adaptor protein co-immunoprecipitation

INS-1 cells were homogenized in a Potter–Elvehjem homogenizer in 125 mmol l^−1^ KCl, 1 mmol l^−1^ EDTA, 50 mmol l^−1^ sucrose, 20 mmol l^−1^ HEPES pH 7.4 and 1% protease inhibitor cocktail (Sigma-Aldrich) and then centrifuged at 2,000*g* for 10 min. The supernatant was used for co-immunoprecipitation with AP1M1, AP2B1 and AP3D1 as described above.

#### Insulin–biotin-pulldown with purified inceptor ECD-His

Insulin–biotin was synthesized as described above and immobilized on Pierce Streptavidin Magnetic Beads (Thermo Fisher Scientific, 88817). His-tagged inceptor ectodomain (pCDNA3-inceptor ECD-6×His) was expressed and purified from HEK293 cells as previously described^29^ and added onto the beads in pH-adjusted lysis buffer (2% NP-40, 1% Triton-X-100, 10% glycerol, 1% protease inhibitor in PBS) at 4 °C overnight. The beads were washed with the pH-adjusted lysis buffer and eluted into Laemmli buffer as described above.

#### Insulin–biotin pulldown from MIN6 cells and C2C12 cells

MIN6 or C2C12 cells were incubated in growth medium or starvation medium (114 mmol l^−1^ NaCl, 4.7 mmol l^−1^ KCl, 1.2 mmol l^−1^ KH_2_PO_4_, 1.16 mmol l^−1^ MgSO_4_, 2.5 mmol l^−1^ CaCl_2_, 25.5 mmol l^−1^ NaHCO_3_, 20 mmol l^−1^ HEPES pH 7.2) for 1 h, followed by incubation with 100 nmol l^−1^ insulin–biotin or 100 nmol l^−1^ insulin in the growth or starvation media. Cells were lysed with 2% NP-40, 1% Triton-X-100, 10% glycerol and 1% protease inhibitor in PBS for 20 min and centrifuged at 21,000*g* for 10 min; then 100 μg of the supernatant was loaded onto Pierce Streptavidin Magnetic Beads at 4 °C overnight. The beads were washed with lysis buffer and eluted in Laemmli buffer as described above.

#### Western blot

Samples were boiled in Laemmli buffer, loaded on a precast 4–20% gradient SDS-polyacrylamide gel (Bio-Rad), separated by electrophoresis and transferred onto a PVDF membrane using a Trans-Blot Turbo (Bio-Rad). The membrane was blocked in 5% milk in TBS-T, incubated with primary antibodies at 4 °C overnight, washed with TBS-T, incubated with HRP-coupled secondary antibodies (1:5,000) for 1 h and then with the Clarity Western ECL substrate (Bio-Rad). Images were acquired with a ChemStudio SA2 (Analytic Jena) and quantified using the Fiji GelAnalyzer (ImageJ v.1.53o)^42^.

### In vitro dimerization and binding assays

Purified inceptor ECD-His peptide was equilibrated to different pH in a mass-spectrometry-compatible buffer made of 150 mM ammonium acetate (from a 7.5 mol l^−1^ stock solution; Sigma-Aldrich) and pH-adjusted to 3, 4, 5, 6 and 7 with formic acid. Then, 25 µg of purified inceptor ECD-His was subjected to buffer exchange in 500 µl spin filters with a 30 kDa cutoff (Vivaspin 500 PES, Sartorius) over five rounds of centrifugation (15 min, 3,000*g* at 4 °C) and resuspension in fresh buffer. Incubation with 0, 30 and 100 µmol l^−1^ human insulin (recombinant; Sigma-Aldrich) or bovine proinsulin (Novo BioLabs) was done in 35 µl final volume (6.5 µmol l^−1^ inceptor) by adding 2 mg ml^−1^ insulin or proinsulin stock solution dissolved in 0.1% formic acid (~pH 2.7). Dilution of the initial 25 µl volumes with 0.1% formic acid did not alter the desired pH (as tested on larger volumes with a pH meter). After incubation for 30 min at 37 °C, samples were directly measured, either by shotgun mass spectrometry or CDMS, or stored for a few days at 4 °C to test binding stability.

For the differential alkylation experiment, inceptor ECD-His solubilized in PBS at pH 7.2 was alkylated with *N*-ethylmaleimide (NEM) for 1 h at room temperature in the dark. After buffer exchange with PBS at pH 7.2, disulphide bonds were reduced by the addition of 8 mM dithiothreitol for 30 min at 54 °C, followed by alkylation with 16 mM IAA for 1 h at room temperature in the dark. The mass spectrometry data were recorded on the Fusion Orbitrap platform connected online to an Ultimate 3000 RSLC nano system through an analytical column (Poroshell EC-C18, 2.7 µm, 50 cm × 75 µm). Peptides were separated over a 60 min gradient with standard shotgun settings applied to the mass spectrometry platform. The resulting data were analysed with MaxQuant^65^ (v.2.0.3.0) with standard settings applied and searched against the full human proteome supplemented with the sequence of inceptor. The intensities were extracted from the NEM and carbamidomethyl site-specific tables and a final ratio was calculated. Peptides in which one C was annotated with NEM and another with carbamidomethyl were excluded from the analysis.

For the insulin and proinsulin binding CDMS assays, samples containing inceptor (5 µM) alone or inceptor with insulin at a ratio of 1:6 and 1:20 and at varying pH values (pH 3–7) were incubated at 37 °C for 30 min before analysis. Free and insulin-bound inceptor samples were introduced into an Orbitrap Q Exactive UHMR mass spectrometer with glass capillaries produced in-house, using the following settings: capillary voltage, 1.5-2.0 kV; ion mode, positive; collision gas, nitrogen; in-source trapping voltage, 50–100 V; noise level, 3.64; number of microscans, 1; injection time, 1–10 ms. The HCD direct eV setting was optimized to ensure sufficient desolvation and cooling of ions and to minimize the number of split peaks observed in the raw CDMS data. The resolution was set to 280,000 at 200 Th (1 s transient time) for all measurements. The calibration of the Orbitrap detector was performed using CsI in the *m*/*z* range of interest (350–12,500 Th). The acquisition was typically performed for 10–20 min for the recording of 1,000–10,000 single ions. The resulting raw data were processed to remove dephasing ions (detected as split peaks) for accurate charge determination^66^. The intensity value cutoff for noise rejection was set to approximately eight to ten elementary charges. All remaining intensities of centroid peaks were normalized to 1 s injection time, and a factor of 12.55 was applied to convert from intensity to charges based on the previous study. Such conversion results in charge determination accuracy within 1.6 elementary charges, which holds true for up to 250 charges and allows for calculation of the final mass. The amount of insulin bound to the monomer and dimer of inceptor was calculated by subtracting the masses of free inceptor (averaged for all pH values) from the masses of inceptor incubated at 1:6 and 1:20 ratios with insulin.

### Preparation and evaluation of inceptor mAB

The purified ectodomain of human inceptor (KIAA1324, Uniprot entry Q6UXG2, residues 1–910) was generated as described previously^29^. A monoclonal rat antibody was generated by immunization with the purified human inceptor ectodomain and validated by immunostaining^67^. The purified ectodomain and rat antibody were kindly provided by Ünal Coskun. The humanized version of the anti-inceptor ectodomain antibody was generated by Yumab. As a control antibody, palivizumab was used.

SC islets were incubated with humanized inceptor mAB or control antibody throughout S5 and S6. The antibody-containing medium was replaced every other day. The mAB uptake was quantified by flow cytometry and immunofluorescence using an anti-human secondary antibody. For the antibody washout experiment, SC islets were washed and the medium was exchanged for fresh S6 medium without mAB. The SC islets were fixed after 0, 2, 6, 24, 48 and 168 h. Whole fixed SC islets were cryo-sectioned for immunostaining and fixed single cells were stained for flow cytometry analysis for insulin and the human antibody.

### Data reporting

No statistical methods were used to pre-determine sample sizes. The experiments were not randomized, and the investigators were not blinded to allocation during experiments and outcome assessment. For the PLA and FRET analysis, the ROUT method was used with the GraphPad Prism software to exclude outliers (*Q* = 1%).

### Statistics and reproducibility

A value of *P* < 0.05 was considered statistically significant. All statistical tests, sample sizes and their *P* values are provided in the figure legends. Unless otherwise specified, statistical tests to compare two groups were performed as two-tailed, and the minimum sample size for representative experiments was *n* = 3. Data distribution was assumed to be normal but this was not formally tested. Unless differently specified, statistics were performed using GraphPad Prism (v.9 or v.10.1.2; GraphPad Software).

### Reporting summary

Further information on research design is available in the Nature Portfolio Reporting Summary linked to this article.

## Supplementary information

Supplementary Information Supplementary Tables 1–6. Supplementary Fig. 1 providing the uncropped blots. Supporting information and compound characterization for insulin derivatives.

Reporting Summary

## Source data

Source Data Fig. 1 Statistical source data.

Source Data Fig. 2 Statistical source data.

Source Data Fig. 3 Statistical source data.

Source Data Fig. 5 Statistical source data.

Source Data Fig. 6 Statistical source data.

Source Data Extended Data Fig./Table 1 Statistical source data.

Source Data Extended Data Fig./Table 3 Statistical source data.

Source Data Extended Data Fig./Table 4 Statistical source data.

Source Data Extended Data Fig./Table 5 Statistical source data.

Source Data Extended Data Fig./Table 7 Statistical source data.

## Data Availability

The scRNA-seq data have been deposited in the Gene Expression Omnibus (GEO) with the accession code GSE226346. The mass spectrometry proteomics data have been deposited to the ProteomeXchange Consortium via the PRIDE partner repository with the dataset identifier PXD040758. Source data are provided with this paper.

## Code availability

Scripts for scRNA-seq analysis are available at https://github.com/lickert-lab/2024_Siehler-Bilekova_Inceptor-KO_SC-islets.
