# Supplementary material for: Inceptor binds to and directs insulin towards lysosomal degradation in β cells
Source: Nat Metab. 2024 Nov 25;6(12):2374–90. doi: 10.1038/s42255-024-01164-y (PMC11659164; doi:10.1038/s42255-024-01164-y)

nature metabolism

Supplementary information https://doi.org/10.1038/s42255-024-01164-y

Inceptor binds to and directs insulin towards lysosomal degradation in β cells

In the format provided by the authors and unedited

Supplementary Table 1 - sgRNA for *IIR* gene targeting

	Target sequence	Strand	Fwd oligonucleotide	Rev oligonucleotide
**1**	GACAACGCTATGGCTG AGCC	Fwd	CACCGGGGACAACGCTATGGC TGAGCC	AAACGGCTCAGCCATAGCGTT GTCCCC
**2**	GAACTGAGAGGCGCA TACCC	Fwd	CACCGGGGAACTGAGAGGCG CATACCC	AAACGGGTATGCGCCTCTCAG TTCCCC
**3**	AAGCTCCGGTCCCGTT CCCT	Rev	CACCGGGAAGCTCCGGTCCCG TTCCCT	AAACAGGGAACGGGACCGGA GCTTCCC
**4**	GGCGCATACCCCGGCT GTGG	Fwd	CACCGGGGGCGCATACCCCGG CTGTGG	AAACCCACAGCCGGGGTATGC GCCCCC

Supplementary Table 2: Differentiation protocol

Differentiation stage (S)	Differentiation day	Basic medium	Growth factors and cytokines
**iPSC**	-3 and -2	StemMACS™ iPS-Brew XF (Miltenyi Biotec, 130-104-368)	Y-27632, 10 µmol/L (SantaCruz, sc-281642A)
**iPSC**	-1	StemMACS™ iPS-Brew XF	-
**S1 (anterior definitive endoderm)**	0	B-S1	Activin A, 100 ng/mL (Peprotech, 120-14-300)<br>CHIR-99021, 5 µmol/L (Tebu-bio, 24804-0004)
**S1 (anterior definitive endoderm)**	1 and 2	B-S1	Activin A, 100 ng/mL
**S2 (primitive gut tube)**	3 to 5	B-S2	FGF7, 50 ng/mL (Peprotech, 100-19-300)
**S3 (pancreatic progenitor 1)**	6	B-S3/4	FGF7, 50 ng/mL<br>LDN, 200 nmol/L (Tebu-bio, T1935)<br>PdBU, 500 nmol/L (Millipore, 524390)<br>SANT-1, 0.25 µmol/L (Sigma-Aldrich, S4572)<br>RA, 2 µmol/L (Sigma-Aldrich, R2625)<br>Y-27632, 10 µmol/L
**S4 (pancreatic progenitor 2)**	7 to 11	B-S3/4	FGF7, 50 ng/mL<br>Activin A, 5 ng/mL<br>SANT-1, 0.25 µmol/L<br>RA, 0.1 µmol/L<br>Y-27632, 10 µmol/L
**S5 (endocrine progenitor)**	12 to 18	B-S5	T3, 1 µmol/L (Sigma-Aldrich, T6397)<br>Alk5iII, 10 µmol/L (Enzo Life Sciences, ALX-270-445-M005)<br>SANT-1, 0.25 µmol/L<br>RA, 0.1 µmol/L<br>BTC, 20 ng/mL (Novus Biologicals, 261-CE-250/CF)<br>GSiXXI, 1 µmol/L (Millipore, 565790)
**S6 (SC-islet)**	19 and 20	B-S6	Y-27632, 10 µmol/L
**S6 (SC-islet)**	21 to 40	B-S6	-

Supplementary Table 3: Basic differentiation media

	**B-S1**	**B-S2**	**B-S3/4**	**B-S5**	**B-S6**
**MCDB131 (Gibco, 10372-019)**	1x	1x	1x	1x	1x
**Glutamax 100x (Gibco, 35050038)**	1x	1x	1x	1x	1x
**BSA fraction V, fatty acid free (Roche, 10775835001)**	2%	2%	2%	2%	2%
**$NaHCO_3$ (Carl Roth, 8551.1)**	43 mmol/L	29 mmol/L	29 mmol/L	35 mmol/L	14 mmol/L
**Glucose (Sigma-Aldrich, G7528)**	8 mmol/L	8 mmol/L	8 mmol/L	25.5 mmol/L	8 mmol/L
**ITS-X 100x (Gibco, 51500-056)**	0.002x	0.002x	0.5x	0.5x	-
**Vitamin C (Sigma-Aldrich, A4544)**	44 mg/L	44 mg/L	44 mg/L	44 mg/L	-
**Penicillin-Streptomycin, 10000 U/mL (Gibco, 15140122)**	100 U/mL	100 U/mL	100 U/mL	100 U/mL	100 U/mL
**Heparin (Sigma-Aldrich, H3149)**	-	-	-	0.01 g/L	0.01 g/L
**Trace elements A 1000x (Corning, 15333641)**	-	-	-	-	1x
**Trace elements B 1000x (Corning, 15343641)**	-	-	-	-	1x
**$ZnSO_4$ (Sigma-Aldrich, Z0251)**	-	-	-	-	1 µmol/L
**MEM Nonessential Amino Acid Solution 100x (Corning, 15333581)**	-	-	-	-	1x

Supplementary Table 4 - Human islet batch and donor overview. Islets from two female and two male donors. Donors had a BMI between 23.5 and 35.8. The HbA1c was unknown for one donor and ranged from 3.8 to 5.8 for the other three donors.

Donor	Age	Preparation purity
**A**	55	85%
**B**	60	95%
**C**	52	90%
**D**	35	95%

Supplementary Table 5: Primary antibodies

Abbreviations: WB: Western blot, co-IP: co-immunoprecipitation, FC: flow cytometry, IF: immunofluorescence, PLA: proximity ligation assay, TEM: transmission electron microscopy

Antigen	Manufacturer	Catalogue number	Application
**Adaptin β (Clone 74)**	BD Biosciences	610382	1:1000 (WB)
**AP1M1**	Invitrogen	PA5-104319	2 µg (co-IP)
**AP2B1**	Abcam	ab205014	2 µg (co-IP)
**AP3D1**	Deposited to the DSHB by Peden, A.A.	anti-delta-SA4	2 µg (co-IP)
**Caspase-3, Cleaved (Asp175)**	Cell Signaling Technology	9661S	1:100 (FC)
**Cathepsin B**	R&D Systems	AF953	1:100 (IF)
**CHGA**	Agilent Dako	M0869	1:200 (IF)
**Clathrin heavy chain**	Cell Signaling Technology	2410	1:100 (IF)
**C-Peptide**	Abcam	ab30477	1:200 (IF)<br>1:100 (FC)
**FOXA2 (HNF-3β) (D56D)**	Cell Signaling Technology	8186S	1:250 (IF)<br>1:200 (FC)
**Glucagon**	Covalab	pab75571	1:600 (IF)
**Glucagon (Clone K79BB10)**	Sigma-Aldrich	G2654-.2ML	1:1000 (IF)<br>1:100 (FC)
**GLG-1**	Novus	AF7879-SP	1:200 (IF)
**Golgin-97**	Cell Signaling Technology	13192S	1:100 (IF)
**Inceptor**	In-house[31] / Yumab	2G6 (rat / humanised)	1 µg/mL (S6 treatment)<br>5 µg/mL (PLA treatment)
**Inceptor**	In-house[31]	16F6 (rat)	1:1000 (IF)<br>1:200 (FC)<br>1:100 (TEM)<br>2 µg (co-IP)
**Inceptor**	In-house[31]	14F1 (rat)	1:1000 (WB)
**Inceptor**	In-house[31]	1374	1:1000 (PLA)
**INSR**	Cell Signaling Technology	3020	1:1000 (WB)
**Insulin**	Bio-Rad	5330-0104G	1:400 (IF)
**Insulin**	Cell Signaling Technology	3014	1:1000 (WB)
**Insulin**	Cell Signaling Technology	8138	1:1000 (WB)

**Insulin**	Sigma	I2018	1:1000 (IF, human tissue)
**LAMP2 / CD107b (Clone H4B4)**	Becton Dickinson	555803	1:100 (IF)<br>1:100 (TEM)
**Mouse IgG control (G3A1)**	Cell Signaling Technology	5415	2 µg (co-IP)
**NKX2-2**	Abcam	ab187375-500ul	1:300 (IF)<br>1:200 (FC)
**NKX6-1**	Deposited to the DSHB by Madsen, O.D	F55A10	1:200 (FC)
**NKX6-1**	Novus	NBP1-82553	1:300 (IF)<br>1:200 (FC)
**PDX1**	R&D Systems	AF2419	1:500 (IF)<br>1:100 (FC)
**Palivizumab**	Yumab	Ypr-2021-49-25	1 µg/mL (S6 treatment)<br>5 µg/mL (PLA treatment)
**Proinsulin**	R&D Systems	MAB13361	1:400 (IF)<br>2 µg (co-IP)<br>1:300 (TEM)<br>1:200 (PLA)
**Rab5**	Cell Signaling Technology	3547S	1:200 (IF)
**Rabbit IgG control (DA1E)**	Cell Signaling Technology	3900	2 µg (co-IP)
**Rat IgG control**	In-house[31]	11A7 (Rat IgG2b)	2 µg (co-IP)
**SOX17**	Neuromics	GT15094	1:400 (IF)<br>1:200 (FC)
**TGN46**	Bio-Rad	AHP500GT	1:200 (IF)
**Tubulin γ**	Sigma-Aldrich	T5326	1:5000 (WB)

Supplementary Table 6: Secondary antibodies

Antibody	Conjugation	Company	Ordering number
**anti-Mouse IgG**	Alexa Fluor 488	Invitrogen	A21202
**anti-Mouse IgG**	Alexa Fluor 555	Invitrogen	A31570
**anti-Mouse**	Alexa Fluor 647	Invitrogen	715-605-151
**anti-Goat IgG**	Alexa Fluor 488	Invitrogen	A11055
**anti-Goat IgG**	Alexa Fluor 555	Invitrogen	A21432
**anti-Goat**	Alexa Fluor 647	Invitrogen	705-605-147
**anti-Rabbit IgG**	Alexa Fluor 488	Invitrogen	A21206
**anti-Rabbit IgG**	Alexa Fluor 555	Invitrogen	A31572
**anti-Rabbit IgG**	Alexa Fluor 647	Invitrogen	A31573
**anti-Rat IgG**	Alexa Fluor 488	Invitrogen	A21208
**anti-Rat IgG**	Cy3	Jackson ImmunoResearch	712-165-153
**anti-Rat IgG**	Alexa Fluor 647	Jackson ImmunoResearch	712-605-150
**anti-Guinea Pig IgG**	Alexa Fluor 488	Jackson ImmunoResearch	706-545-148
**anti-Guinea Pig IgG**	Cy3	Jackson ImmunoResearch	706-165-148
**anti-Guinea Pig IgG**	Alexa Fluor 647	Jackson ImmunoResearch	706-495-148
**anti-Sheep IgG**	Alexa Fluor 488	Jackson ImmunoResearch	713-546-147
**anti-Sheep IgG**	Alexa Fluor 555	Invitrogen	A21436
**anti-Sheep IgG**	Alexa Fluor 647	Jackson ImmunoResearch	713-606-147
**anti-Mouse IgG**	Alexa Fluor 594	Jackson ImmunoResearch	115 585 003
**anti-Rat IgG**	Alexa Fluor 488	Invitrogen	A11006
**anti-Mouse IgG (H+L)**	HRP	Jackson ImmunoResearch	115-035-146
**anti-Rabbit IgG (H+L)**	HRP	Jackson ImmunoResearch	111-035-144
**anti-Rat IgG, Light Chain spec.**	HRP	Jackson ImmunoResearch	112-035-175
**anti-Rat IgG**	12nm gold	Jackson Immuno Research	112-205-143
**anti-Mouse IgG**	6nm gold	Jackson Immuno Research	115-195-166
**Anti-Human IgG**	Alexa Fluor 488	Invitrogen	A11013
**Anti-Human IgG**	Alexa Fluor 647	Invitrogen	A21445
**Rabbit anti-Mouse IgG**	-	Bridging AB for TEM	Slot et al, Nat. Protoc., 2007
**Rabbit anti-Rat IgG**	-	Bridging AB for TEM	Slot et al, Nat. Protoc., 2007

Supplementary Fig. 1 Uncropped western blots

Fig. 4e

a) Inceptor

Membrane photograph

kDa
250
130

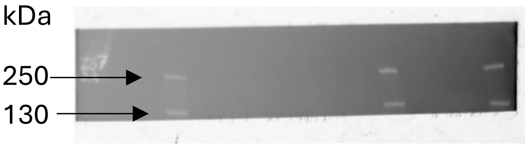

Developed blot

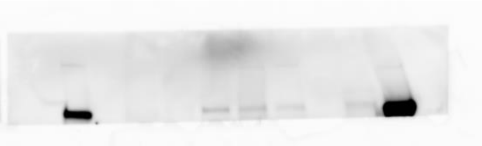

b) β-adaptin

Membrane photograph

kDa
100
70

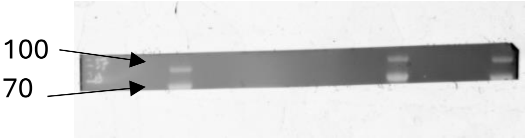

Developed blot

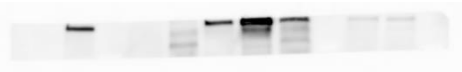

Fig. 5e

a) inceptor – membrane photograph and developed blot overlay

kDa
130
100

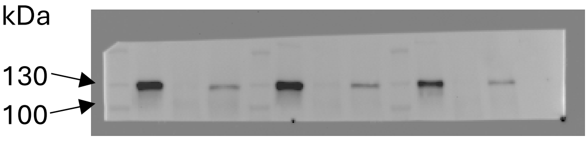

b) (pro)insulin

kDa
15
10

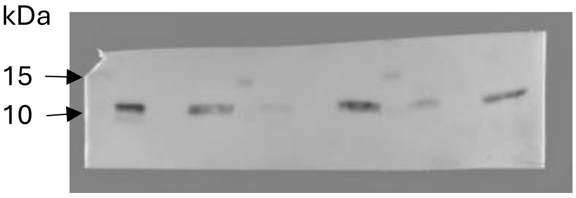

Extended Data Fig. 7b

Pulldown

kDa
130

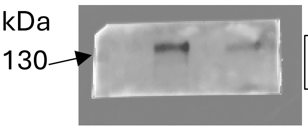

inceptor

100

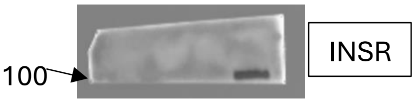

INSR

Input

kDa
130

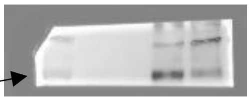

inceptor

100

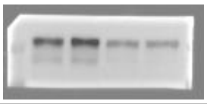

INSR

55

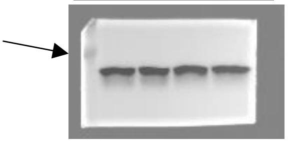

γ-tubulin

Extended Data Fig. 7d

a) Inceptor

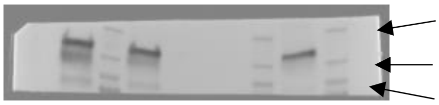

kDa
250
100
75

b) γ-tubulin

55
35
25

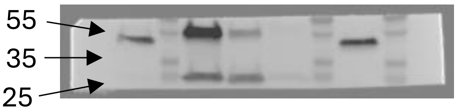

c) (pro)insulin

kDa
10

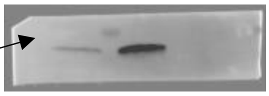

Extended Data Fig. 7e

a) inceptor

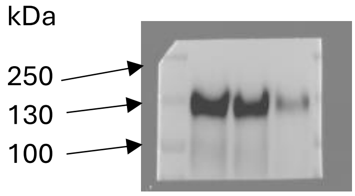

b) proinsulin

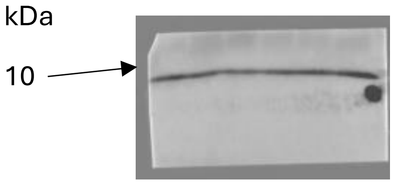

Methods

Concentration of samples:

Amicon Ultra filters from Merck millipore were used to concentrate aqueous samples.

LC-MS:

Insulin molecules were measured on a Waters Alliance HT 2795 system coupled with a Waters Micromass LCT-Premier MS detector using a C18 wide pore column (ESI-MS$^+$): 0 min, 99% H_2O (0.05% TFA) / 1% acetonitrile (0.05% TFA) → 5 min 10 % H_2O ((0.05% TFA) / 90% acetonitrile (0.05% TFA); 0.6 ml / min; 30 °C).

HR-MS:

High-resolution mass spectra were measured on a Water Aquity UPLC system with a QTof Premier detector (ESI and APCI-MS/MS) or on a Water Allicance 2695 with Micromass LCTPremier detector. Samples were dissolved in deion. H_2O and injected using either an HPLC system or a direct inlet. For insulin samples, a peptide column (ACQUITY UPLC BEH300 C4 or C18 1.7 µm) was used in the HPLC system. The measured values are given in mass/charge (m/z).

HPLC

Column chromatography was carried out using an automated Reveleris Prep. Plant system from Buchi. For insulin molecules a RP-peptide column (Jupiter® 10 µm Proteo 90 Å, LC Column 250 x 30 mm from Phenomenex) were used. The gradient is given below (Solvent A: H_2O + 0.05 % TFA, Solvent B: acetonitrile + 0.05 % TFA).

Gradient Table

	Min	Solvents	% 2nd
1	0.0	AB	10
2	5.0	AB	20
3	5.0	AB	20
4	5.0	AB	30
5	5.0	AB	30
6	35.0	AB	55
7	0.0	AB	90
8	5.0	AB	90
9	0.0	AB	10
10	7.0	AB	10

NMR spectra

NMR spectra were recorded on Bruker spectrometers. The measurements were made on DPX-400 (^1H: 400.13 MHz), at room temperature and in the respective deuterated

solvents indicated. The residual proton signal of the solvent used ($CDCl_3$: δ (^{1}H-NMR) = 7.26 ppm, is used as a reference and to calibrate the ^{1}H-NMR spectra. Coupling constants *J* were expressed in Hz and chemical shifts δ in ppm. Signal multiplicities were abbreviated as follows for simplicity: singlet (s), duplet (d), triplet (t), quartet (q), and multiplet (m).

Compound Characterization

Insulin-Azide

LC-MS (DEFAULT 01-90 ACN): t_R = 2.69 min, m/z (ESI-MS$^+$) for $C_{261}H_{391}O_{78}N_{68}S_6^{3+}$ $(M+3H)^{3+}$ = 1974.

HR-Mass: calculated m/z für $C_{261}H_{393}O_{78}N_{68}S_6^{5+}$ $(M+5H)^{5+}$ = 1183.9440, found (ESI-MS$^+$): 1183.9456.

LC-MS chromatogram and mass spectrum of **Insulin-azide** (m/z (ESI-MS$^+$) = 1185 $(M+5H)^{5+}$, 1481 $(M+4H)^{4+}$, 1974 $(M+3H)^{3+}$).

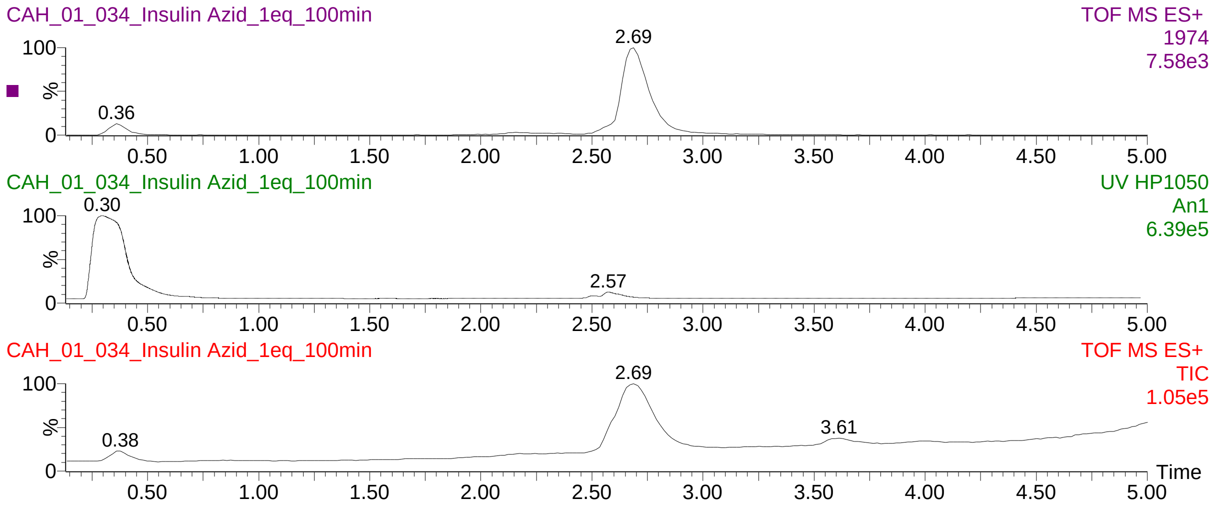

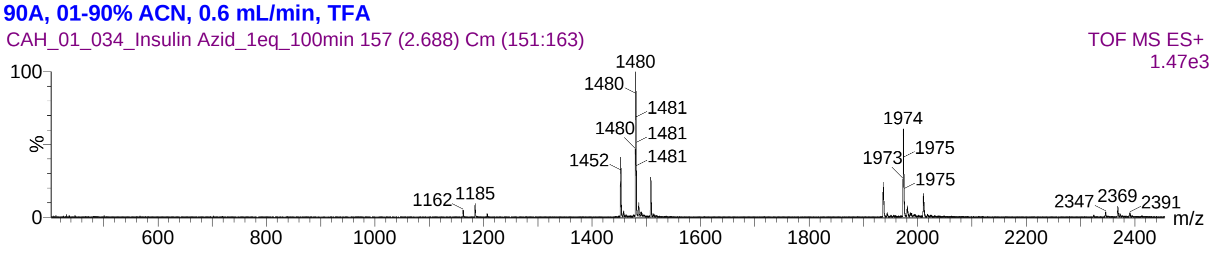

Insulin-Bodipy 630/650 (Ins-630)

LC-MS (DEFAULT 01-90 ACN): t_R = 3.01 min, m/z (ESI-MS$^+$) for $C_{287}H_{412}BF_2N_{71}O_{80}S_7^{4+}$ $(M+4H)^{4+}$ = 1602.

LC-MS chromatogram and mass spectrum of **Ins-BDP630/650** (m/z (ESI-MS$^+$) = 2136 $(M+3H)^{3+}$, 1602 $(M+4H)^{4+}$ and 1282 $(M+5H)^{5+}$).

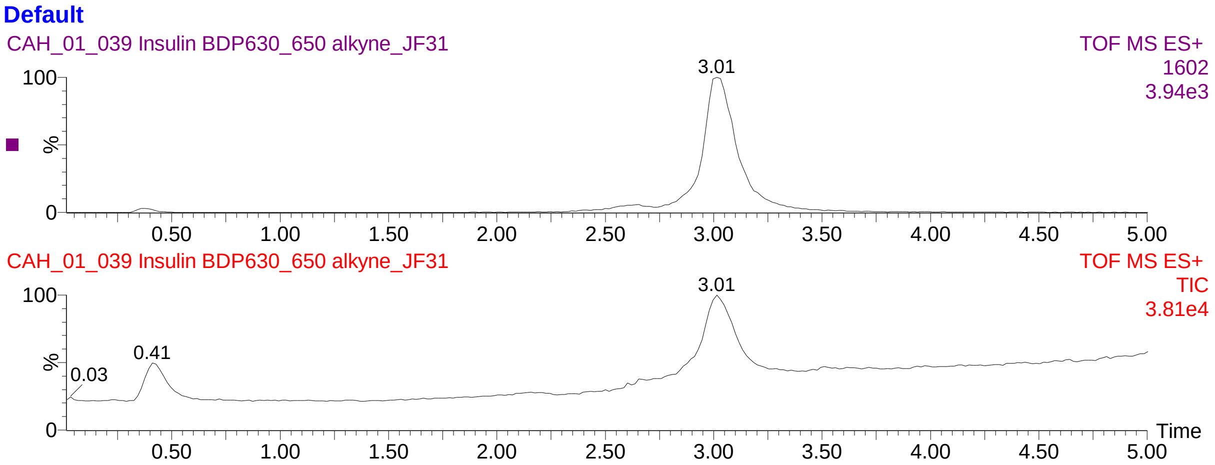

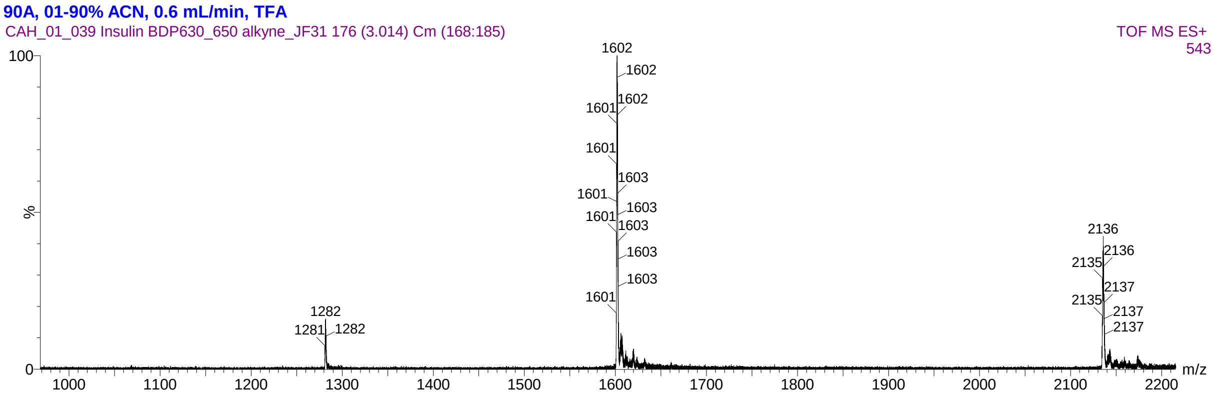

Biotin-PEG(9)-azide

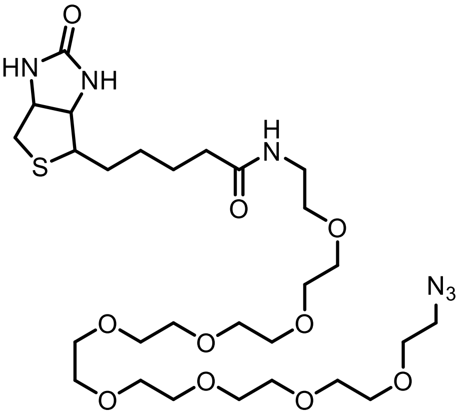

HR-ESI-MS: calculated for $C_{28}H_{52}N_6O_{10}SNa^+$: 687.3363 $[M+Na]^+$, found: 687.3351.

^{1}H-NMR (400 MHz, $CDCl_3$) δ = 6.57 (t, *J* = 5.2 Hz, 1H), 5.97 (s, 1H), 5.10 (s, 1H), 4.50 (dd, *J* = 5.1, 7.6 Hz, 1H), 4.32 (t, *J* = 5.8 Hz, 1H), 3.67-3.63 (m, 33H), 3.45-3.42 (m, 2H), 3.39 (t, *J* = 5.1 Hz, 2H), 3.17-3.12 (m, 1H), 2.91 (dd, *J* = 4.9, 12.8 Hz, 1H), 2.74 (d, *J* = 12.7 Hz, 1H), 2.26-2.18 (m, 2H), 1.78-1.62 (m, 3H), 1.49-1.41 (m, 2H).

^{1}H-NMR spectra (400 MHz, $CDCl_3$) of Biotin-PEG(9)-azide

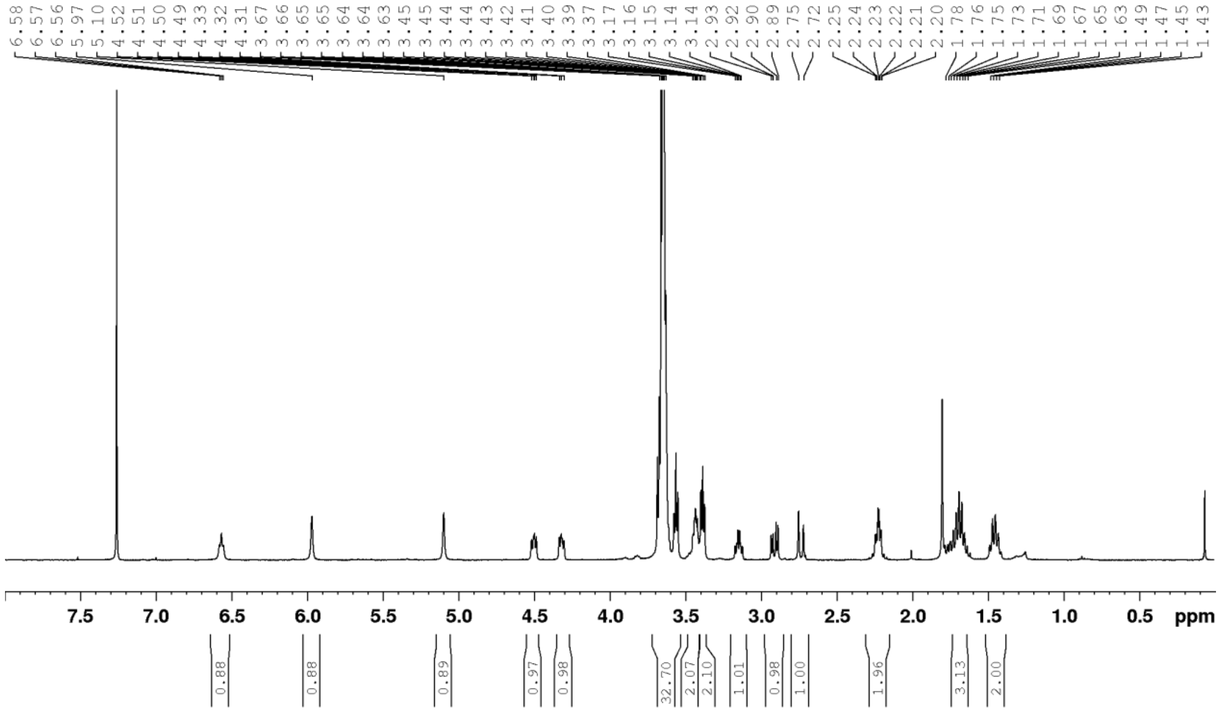

Insulin-Biotin

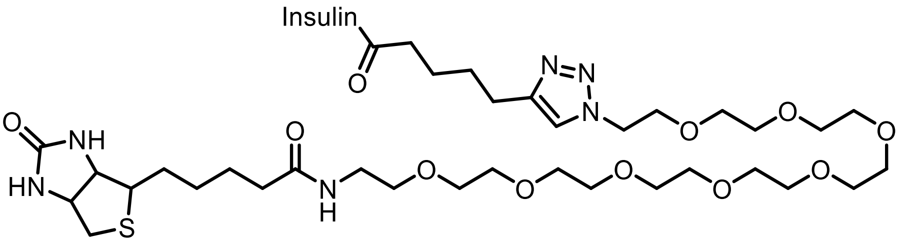

LC-MS (DEFAULT 01-90 ACN): t_R = 2.60 min, m/z (ESI-MS$^+$) for $C_{292}H_{448}N_{71}O_{88}S_7^{4+}$ $(M+4H)^{4+}$ = 1646.

LC-MS chromatogram, UV trace (224 nm) und mass spectrum of Insulin-Biotin (m/z (ESI-MS$^+$) = 1097 $(M+6H)^+$, 1317 $(M+5H)^+$, 1646 $(M+4H)^+$.

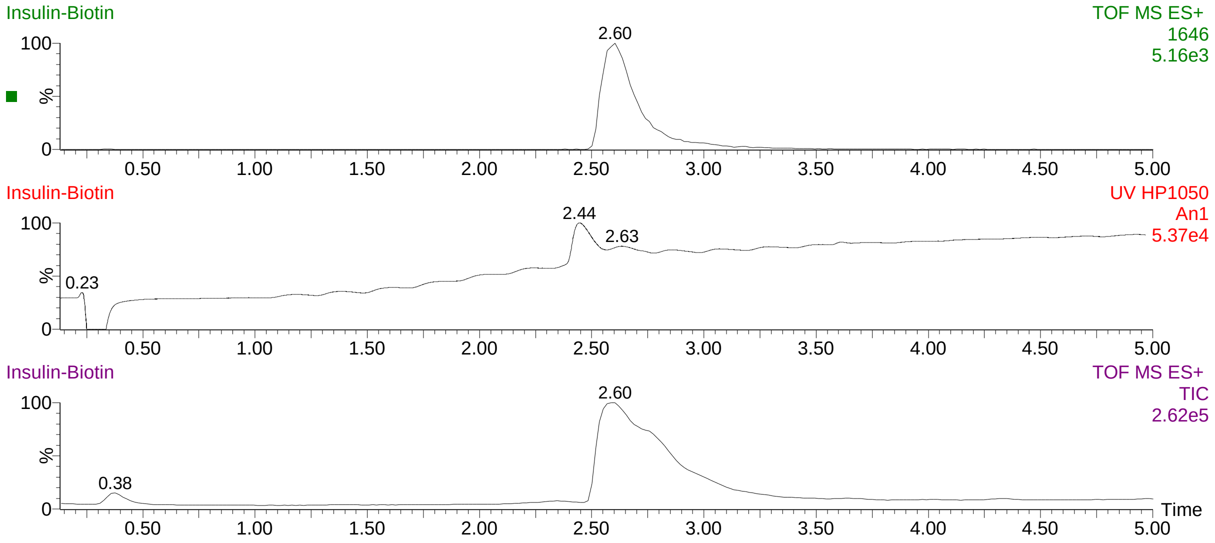

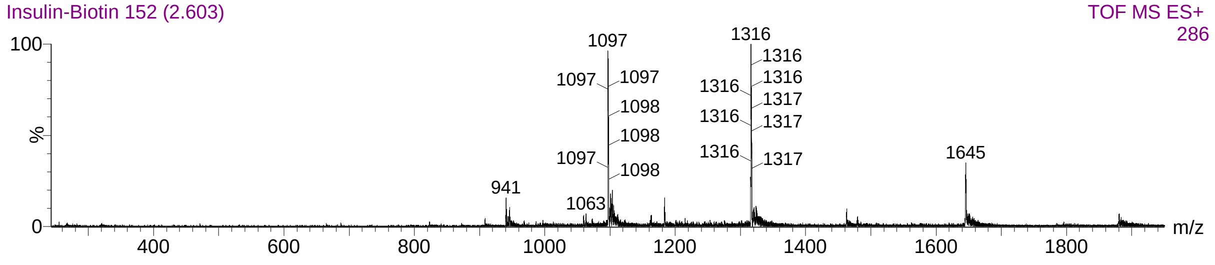

Supplement: Supplementary file 1 — Supplementary Tables 1–6. Supplementary Fig. 1 providing the uncropped blots. Supporting information and compound characterization for insulin derivatives. [file 42255_2024_1164_MOESM1_ESM.pdf]
