# Supplementary material for: Inceptor binds to and directs insulin towards lysosomal degradation in β cells
Source: Nat Metab. 2024 Nov 25;6(12):2374–90. doi: 10.1038/s42255-024-01164-y (PMC11659164; doi:10.1038/s42255-024-01164-y)
Supplement: Supplementary file 2 — Reporting Summary [file 42255_2024_1164_MOESM2_ESM.pdf]

nature portfolio

Corresponding author(s): Heiko Lickert

Last updated by author(s): Sep 3, 2024

Reporting Summary

Nature Portfolio wishes to improve the reproducibility of the work that we publish. This form provides structure for consistency and transparency in reporting. For further information on Nature Portfolio policies, see our Editorial Policies and the Editorial Policy Checklist.

Statistics

For all statistical analyses, confirm that the following items are present in the figure legend, table legend, main text, or Methods section.

n/a	Confirmed	
☐	☒	The exact sample size (*n*) for each experimental group/condition, given as a discrete number and unit of measurement
☐	☒	A statement on whether measurements were taken from distinct samples or whether the same sample was measured repeatedly
☐	☒	The statistical test(s) used AND whether they are one- or two-sided<br>*Only common tests should be described solely by name; describe more complex techniques in the Methods section.*
☒	☐	A description of all covariates tested
☐	☒	A description of any assumptions or corrections, such as tests of normality and adjustment for multiple comparisons
☐	☒	A full description of the statistical parameters including central tendency (e.g. means) or other basic estimates (e.g. regression coefficient) AND variation (e.g. standard deviation) or associated estimates of uncertainty (e.g. confidence intervals)
☐	☒	For null hypothesis testing, the test statistic (e.g. *F*, *t*, *r*) with confidence intervals, effect sizes, degrees of freedom and *P* value noted<br>*Give P values as exact values whenever suitable.*
☒	☐	For Bayesian analysis, information on the choice of priors and Markov chain Monte Carlo settings
☒	☐	For hierarchical and complex designs, identification of the appropriate level for tests and full reporting of outcomes
☒	☐	Estimates of effect sizes (e.g. Cohen's *d*, Pearson's *r*), indicating how they were calculated

Our web collection on statistics for biologists contains articles on many of the points above.

Software and code

Policy information about availability of computer code

Data collection	Zen Blue 3.9 (Zeiss), Wasabi software 1.5 (Hamamatsu Photonics)
Data analysis	Zen Blue 3.9 (Zeiss), Fiji (ImageJ 1.53o), FlowJo™ v10.7.1, CellRanger software (10x Genomics) (v3.1.0), DropletUtils(v1.14.2), scrublet (v0.2.3), DoubletDetection (v4.2), scds (v1.10.0), scDblFinder(1.11.4), DoubletFinder (v2.0.3) and SOLO (implemented in scvi-tools v0.17.1), sctransform (v0.3.3), Seurat (v4.1.1), harmonypy (v0.0.6), QuPath software (version 0.4.3), GraphPad Prism 10.1.2, MaxQuant v2.0.3.0.

For manuscripts utilizing custom algorithms or software that are central to the research but not yet described in published literature, software must be made available to editors and reviewers. We strongly encourage code deposition in a community repository (e.g. GitHub). See the Nature Portfolio guidelines for submitting code & software for further information.

Data

Policy information about availability of data

All manuscripts must include a data availability statement. This statement should provide the following information, where applicable:

- Accession codes, unique identifiers, or web links for publicly available datasets
- A description of any restrictions on data availability
- For clinical datasets or third party data, please ensure that the statement adheres to our policy

The scRNA-seq data have been deposited at the GEO with the accession code GSE226346. The mass spectrometry proteomics data have been deposited to the ProteomeXchange Consortium via the PRIDE partner repository with the dataset identifier PXD040758. Source data will be provided upon publication.

Research involving human participants, their data, or biological material

Policy information about studies with human participants or human data. See also policy information about sex, gender (identity/presentation), and sexual orientation and race, ethnicity and racism.

Reporting on sex and gender	Sex and gender were not considered in the selection of tissues.
Reporting on race, ethnicity, or other socially relevant groupings	Race and ethnicity were not considered in the selection of tissues.
Population characteristics	No covariates were used for population characteristic.
Recruitment	Human islets for research were provided by the Alberta Diabetes Institute IsletCore at the University of Alberta in Edmonton (www.bcell.org/adi-isletcore) with the assistance of the Human Organ Procurement and Exchange (HOPE) program, Trillium Gift of Life Network (TGLN) and other Canadian organ procurement organizations. Human foetal pancreatic tissues (11-12 weeks post-conception) were provided by the INSERM's HuDeCA Biobank. Maternal written consent was obtained, along with approval from the French agency for biomedical research (Saint-Denis La Plaine, France).
Ethics oversight	Human islets for research were provided by the Alberta Diabetes Institute IsletCore at the University of Alberta in Edmonton (www.bcell.org/adi-isletcore) with the assistance of the Human Organ Procurement and Exchange (HOPE) program, Trillium Gift of Life Network (TGLN) and other Canadian organ procurement organizations. Islet isolation was approved by the Human Research Ethics Board at the University of Alberta (Pro00013094). All donors' families gave informed consent for the use of pancreatic tissue in research. The Ethics Committee of the Technical University Munich positively voted on research on human material (557/16 S).<br><br>Human foetal pancreatic tissues (11-12 weeks post-conception) were provided by the INSERM's HuDeCA Biobank. Maternal written consent was obtained, along with approval from the French agency for biomedical research (Saint-Denis La Plaine, France). Human adult pancreases were harvested from adult brain-dead organ donors before cardiac death with prior consent for research use at the Hôpital Saint-Louis (Paris, France). Human pancreatic tissue was processed in accordance with the French bioethics legislation and INSERM guidelines (French bylaw, published on December 29, 1998).

Note that full information on the approval of the study protocol must also be provided in the manuscript.

Field-specific reporting

Please select the one below that is the best fit for your research. If you are not sure, read the appropriate sections before making your selection.

☒ Life sciences ☐ Behavioural & social sciences ☐ Ecological, evolutionary & environmental sciences

For a reference copy of the document with all sections, see nature.com/documents/nr-reporting-summary-flat.pdf

Life sciences study design

All studies must disclose on these points even when the disclosure is negative.

Sample size	Sample sizes were not calculated in advance. Typically used sample sizes of 3-6 for independent experiments have been used. For single-cell experiments, typically used sample sizes were analyzed.
Data exclusions	For the PLA and the FRET analysis, the ROUT method was used with the GraphPad Prism software to exclude outliers (Q = 1%). For other experiments, no data have been excluded.
Replication	Minimum sample size for representative experiments was n = 3. All attempts at replication were successful.
Randomization	Samples have not been randomized but all samples have been treated equally, analyzed by objective methods (FACS, ELISA, intensity quantification, etc.) and compared to experimental controls.
Blinding	Researchers have not been blinded due to time constraints but ll samples have been treated equally, analyzed by objective methods (FACS, ELISA, intensity quantification, etc.) and compared to experimental controls.

Reporting for specific materials, systems and methods

We require information from authors about some types of materials, experimental systems and methods used in many studies. Here, indicate whether each material, system or method listed is relevant to your study. If you are not sure if a list item applies to your research, read the appropriate section before selecting a response.

Materials & experimental systems

n/a	Involved in the study
☐	☒ Antibodies
☐	☒ Eukaryotic cell lines
☒	☐ Palaeontology and archaeology
☐	☒ Animals and other organisms
☒	☐ Clinical data
☒	☐ Dual use research of concern
☒	☐ Plants

Methods

n/a	Involved in the study
☒	☐ ChIP-seq
☐	☒ Flow cytometry
☒	☐ MRI-based neuroimaging

Antibodies

Antibodies used

Antigen Manufacturer Catalogue number Application
Adaptin β (Clone 74) BD Biosciences 610382 1:1000 (WB)
AP1M1 Invitrogen PA5-104319 1:100 (co-IP)
AP2B1 Abcam ab205014 1:100 (co-IP)
AP3D1 Deposited to the DSHB by Peden, A.A. anti-delta-SA4 1:100 (co-IP)
Caspase-3, Cleaved (Asp175) Cell Signaling Technology 9661S 1:100 (FC)
Cathepsin B R&D Systems AF953 1:100 (IF)
CHGA Agilent Dako M0869 1:200 (IF)
Clathrin heavy chain Cell Signaling Technology 2410 1:100 (IF)
C-Peptide Abcam ab30477 1:200 (IF), 1:100 (FC)
FOXA2 (HNF-3β) (D56D) Cell Signaling Technology 8186S 1:250 (IF), 1:200 (FC)
Glucagon Covalab pab75571 1:600 (IF)
Glucagon (Clone K79BB10) Sigma-Aldrich G2654-.2ML 1:1000 (IF), 1:100 (FC)
GLG-1 Novus AF7879-SP 1:200 (IF)
Golgin-97 Cell Signaling Technology 13192S 1:100 (IF)
Inceptor In-house27 / Yumab 2G6 (rat / humanised) 1 µg/mL (S6 treatment), 5 µg/mL (PLA treatment)
Inceptor In-house27 16F6 (rat) 1:1000 (IF), 1:200 (FC), 1:100 (TEM), 1:100 (co-IP)
Inceptor In-house27 14F1 (rat) 1:1000 (WB)
Inceptor In-house27 1374 1:1000 (PLA)
INSR Cell Signaling Technology 3020 1:1000 (WB)
Insulin Bio-Rad 5330-0104G 1:400 (IF)
Insulin Cell Signaling Technology 3014 1:1000 (WB)
Insulin Cell Signaling Technology 8138 1:1000 (WB)
Insulin Sigma I2018 1:1000 (IF, human tissue)
LAMP2 / CD107b (Clone H4B4) Becton Dickinson 555803 1:100 (IF), 1:100 (TEM)
Mouse IgG control (G3A1) Cell Signaling Technology 5415 1:100 (co-IP)
NKX2-2 Abcam ab187375-500ul 1:300 (IF), 1:200 (FC)
NKX6-1 Deposited to the DSHB by Madsen, O.D F55A10 1:200 (FC)
NKX6-1 Novus NBP1-82553 1:300 (IF), 1:200 (FC)
PDX1 R&D Systems AF2419 1:500 (IF), 1:100 (FC)
Palivizumab Yumab Ypr-2021-49-25 1 µg/mL (S6 treatment), 5 µg/mL (PLA treatment)
Proinsulin R&D Systems MAB13361 1:400 (IF), 1:100 (co-IP), 1:300 (TEM), 1:200 (PLA)
Rab5 Cell Signaling Technology 3547S 1:200 (IF)
Rabbit IgG control (DA1E) Cell Signaling Technology 3900 1:100 (co-IP)
Rat IgG control In-house27 11A7 (Rat IgG2b) 1:100 (co-IP)
SOX17 Neuromics GT15094 1:400 (IF), 1:200 (FC)
TGN46 Bio-Rad AHP500GT 1:200 (IF)
Tubulin γ Sigma-Aldrich T5326 1:5000 (WB)

Validation

All primary antibodies have been validated against an isotype control and for their size by western blot or for their subcellular localization by immunostaining. Commercially available antibodies have validation data available on their website available under the respective catalogue number. Inceptor antibodies have been validated in knockout cell lines previously (Ansarullah et al., 2021, Nature).

Eukaryotic cell lines

Policy information about cell lines and Sex and Gender in Research

Cell line source(s)

iPSC HMGUi001-A (Wang et al., 2018, Molecular Metabolism, obtained from Dr. Wang) female, HMGUi001-A-8 (Siehler et al., 2021, Stem Cell Res., obtained from Dr. Siehler) female, INS-1 (Addex Bio C0018007), INS-1E (C0018009, AddexBio), HEK293 (ATCC CRL-1573), 293T (ATCC CRL-3216), MIN6 K8 (obtained from Jun-ichi Miyazaki, Osaka University), C2C12 (ATCC CRL-1772)

Authentication

iPSC HMGUi001-A and HMGUi001-A-8 cell lines were authenticated by morphology analysis, karyotyping, PCR, and STR profiling. The other cell lines were authenticated by morphology.

Mycoplasma contamination	all cell lines were regularly tested negative for mycoplasma
Commonly misidentified lines (See ICLAC register)	no commonly misidentified cell lines were used.

Animals and other research organisms

Policy information about studies involving animals; ARRIVE guidelines recommended for reporting animal research, and Sex and Gender in Research

Laboratory animals	C57BL/6J wild type and inceptor knockout mice were used for pancreatic tissue. Tissue was collected at 4 months of age.
Wild animals	no wild animals were used.
Reporting on sex	For cell-based analysis, the sex of the donor animal was not considered.
Field-collected samples	the study did not involve samples from the field.
Ethics oversight	Animal experiments were carried out in compliance with the German Animal Protection Act and with the approved guidelines of the Society of Laboratory Animals (GV-SOLAS) and of the Federation of Laboratory Animal Science Associations (FELASA).

Note that full information on the approval of the study protocol must also be provided in the manuscript.

Plants

Seed stocks	NA
Novel plant genotypes	NA
Authentication	NA

Flow Cytometry

Plots

Confirm that:

☒ The axis labels state the marker and fluorochrome used (e.g. CD4-FITC).

☒ The axis scales are clearly visible. Include numbers along axes only for bottom left plot of group (a 'group' is an analysis of identical markers).

☒ All plots are contour plots with outliers or pseudocolor plots.

☒ A numerical value for number of cells or percentage (with statistics) is provided.

Methodology

Sample preparation	differentiating human IPS cells and SC-islets were used for flow cytometry. The clusters were dissociated with Accutase and if applicable, single cells were fixed in PFA.
Instrument	BD FACSAria III
Software	FlowJo
Cell population abundance	Enrichment was confirmed by fluorescence microscopy
Gating strategy	Negative control samples were generated by pooling experimental samples. Isotype control antibodies or no primary antibodies were used to define negative populations.

☒ Tick this box to confirm that a figure exemplifying the gating strategy is provided in the Supplementary Information.
